# Dynamic changes in regional ventilation distribution and end-expiratory lung impedance using electrical impedance tomography: associations with weaning and extubation outcomes

**DOI:** 10.3389/fmed.2026.1904503

**Published:** 2026-10-07

**Authors:** Minjia Wang, Changyun Zhao, Zhiheng Hao, Liang Wu, Wenchao Mao, Jiaping Zhao

**Affiliations:** Department of Critical Care Medicine, Zhejiang Hospital, Hangzhou, Zhejiang, China

**Keywords:** electrical impedance tomography, end-expiratory lung impedance, extubation outcome, rapid shallow breathing index, regional ventilation, spontaneous breathing trial, weaning failure

## Abstract

**Background:**

Accurately assessing the optimal timing for extubation remains a significant challenge in intensive care units (ICUs). The rapid shallow breathing index (RSBI) is widely used but lacks insight into regional lung physiology. This study aimed to characterize dynamic changes in regional ventilation distribution and end-expiratory lung impedance (EELI) using electrical impedance tomography (EIT) during spontaneous breathing trials (SBTs) and to explore their association with weaning and extubation outcomes.

**Methods:**

In this prospective observational study, 52 mechanically ventilated patients undergoing their first SBT were enrolled. EIT parameters, including the Global Inhomogeneity (GI) index, center of ventilation (COV), ventrodorsal ventilation asymmetry (|ROI V-D|), and changes in EELI (ΔEELI), were recorded at four time points. Weaning failure was defined as SBT failure, or as reintubation or noninvasive ventilation within 48 h after extubation. Extubation failure was defined as reintubation or rescue NIV within 48 h. Logistic models coded 1 = failure and 0 = success.

**Results:**

SBT failure (*n* = 19, 36.5%) was associated with progressive deterioration of ventilation, with a significant increase in the GI index (*p* < 0.001) and a notable spike in |ROI V-D| at T3 (*p* = 0.005). Extubation failure (*n* = 9, 27.3% of SBT successes) was associated with a greater cumulative loss of end-expiratory lung volume (ΔEELI T4–T1) compared with the success group (*p* < 0.001). In secondary exploratory analyses, a combined MLE model of T4 RSBI and T3 GI showed an apparent AUC of 0.923 (95% CI 0.840–0.986) for SBT failure (optimism-corrected AUC: 0.905). A combined model of T4 RSBI and ΔEELI (T4–T1) showed an apparent AUC of 0.875 (95% CI: 0.688–0.988) for extubation failure; with only 9 events, this estimate is hypothesis-generating and requires external validation.

**Conclusions:**

EIT-derived parameters offer insights into regional ventilation changes during the weaning process. Increased ventilation inhomogeneity and ventrodorsal mismatch are associated with SBT failure, while loss of end-expiratory lung impedance is associated with extubation failure. Adding EIT parameters to RSBI provided exploratory incremental information for discriminating SBT failure and extubation failure in this cohort. These secondary analyses require external validation before any clinical application can be considered.

**Clinical Trial Registration**: https://www.chictr.org.cn, identifier ChiCTR2500108219.

## Background

Weaning from mechanical ventilation is a critical challenge in intensive care units (ICUs), as prolonged weaning or failure to wean can lead to severe complications, including ventilator-associated pneumonia, airway injury, prolonged ICU stays, and increased mortality rates (1–3). Therefore, accurately assessing the optimal timing for weaning and identifying patients at high risk of weaning failure are essential for improving clinical outcomes (4, 5).

Currently, the rapid shallow breathing index (RSBI) remains the most widely used clinical indicator for predicting weaning outcomes (6), as it evaluates a patient's spontaneous respiratory capacity by calculating the ratio of respiratory rate to tidal volume. However, RSBI only reflects overall respiratory mechanics and cannot provide dynamic information on regional ventilation distribution, lung re-expansion status, or end-expiratory lung volume, which limits its predictive accuracy (7–9).

Electrical impedance tomography (EIT) is a bedside, non-invasive, radiation-free dynamic pulmonary monitoring technique that enables real-time assessment of ventilation distribution, end-expiratory lung impedance (EELI), and regional ventilation inhomogeneity (GI) (10–12). EIT has been widely applied in lung re-expansion assessment and ventilator parameter optimization for patients with acute respiratory distress syndrome (ARDS) (10, 11). Key EIT-derived indices used in this study include the Global Inhomogeneity (GI) index and the Center of Ventilation (COV). The GI index quantifies the spatial heterogeneity of tidal ventilation distribution: it is calculated by determining the median impedance change across all pixels in a tidal variation image, then computing the sum of absolute differences between this median and each pixel's impedance value, divided by the total sum of impedance values. A GI value of approximately 0.45–0.55 is considered normal in healthy lungs, while higher values indicate greater ventilation inhomogeneity. The GI index has been shown to correlate strongly with the degree of regional lung recruitment.

The COV represents the geometric center of ventilation distribution along the ventrodorsal axis, expressed as a percentage. A value of 50% indicates equal ventilation distribution between ventral and dorsal regions; values below 50% indicate a shift toward dorsal (gravity-dependent) lung zones. The COV provides a simple, intuitive measure of whether ventilation is predominantly distributed to dependent or non-dependent lung regions, which has important implications for assessing lung aeration and the risk of derecruitment.

These EIT parameters have demonstrated clinical value in guiding ventilator settings in ARDS, assessing lung recruitment, and optimizing PEEP titration. However, their role in predicting weaning outcomes remains under-investigated, forming the rationale for the present study.

Accordingly, the primary objective of this study was to characterize the temporal evolution of prespecified EIT-derived parameters—Global Inhomogeneity (GI) index, Center of Ventilation (COV), regional ventilation distribution, and end-expiratory lung impedance (EELI)—during spontaneous breathing trials (SBT) in mechanically ventilated patients. The secondary, exploratory objective was to evaluate the association of these EIT parameters with SBT failure and extubation outcomes, and to explore whether combining EIT parameters with the Rapid Shallow Breathing Index (RSBI) could improve predictive performance. All prediction analyses are explicitly designated as exploratory and require external validation.

## Methods

### Study design

This prospective observational cohort study was conducted from September 2025 to March 2026 across two general ICUs at a tertiary hospital in Zhejiang Province, China. The study protocol was approved by the Institutional Ethics Committee (Protocol No.: ZJHIRB-2025-146K) and was performed in accordance with the Declaration of Helsinki. Written informed consent was obtained from all participants with the capacity to provide consent at the time of enrollment. For patients who lacked the capacity to consent due to the severity of acute critical illness, informed consent was obtained from their legally authorized representatives (LARs) before participation. In accordance with the requirements of the Institutional Ethics Committee, participants who regained decision-making capacity during the study period were provided with a full explanation of the study and signed a written re-consent form.

### Study population

Patients receiving invasive mechanical ventilation (MV) for more than 48 h (12) were eligible for inclusion if they met all the following criteria for a SBT (13, 14): resolution or improvement of the underlying cause of MV; adequate oxygenation (SaO_2_ > 90% with fraction of inspired oxygen [FiO_2_] ≤ 0.5, or partial pressure of oxygen in arterial blood [PaO_2_]/FiO_2_ ≥ 150 mmHg with positive end-expiratory pressure [PEEP] ≤ 8 cmH_2_O); favorable pulmonary function (respiratory rate <30 breaths/min, tidal volume ≥5 mL/kg ideal body weight [IBW], and no significant respiratory acidosis); hemodynamic stability (systolic blood pressure 90–160 mmHg, no or minimal vasopressor support, and heart rate <120 beats/min); being awake without sedation; no excessive tracheobronchial secretions; and an effective cough reflex.

Exclusion criteria included age <18 years, pregnancy, thoracic drainage, pneumothorax, or pneumomediastinum, flail chest or rib fractures, cervical spinal cord injury or neuromuscular disease, tracheostomy, implanted pacemaker or defibrillator, chest skin lesions affecting electrode placement, incomplete clinical data, and refusal of participation.

### Study procedures

Eligible patients underwent a 30 min SBT using pressure-support ventilation (PSV) at 5 cmH_2_O with zero PEEP (12). FiO_2_ was maintained at ≤0.5. After a successful SBT, the attending physician, blinded to the EIT parameters, decided to extubate. We chose zero PEEP during SBT for two reasons. First, zero PEEP during SBT reflects the physiological conditions immediately after extubation, when positive pressure is no longer applied. This approach is intended to identify patients who can maintain adequate lung aeration without positive pressure support—a key determinant of successful extubation. Second, zero PEEP during SBT is commonly used in clinical practice and in several large weaning trials, facilitating comparability with existing literature. This choice is also consistent with the recent review by Thille and Hernández, which emphasized that SBT protocols should be standardized but acknowledged that the optimal PEEP level during SBT remains an area of active investigation (15).

Automatic tube compensation (ATC) was not used during the SBT. All patients received pressure-support ventilation at 5 cmH_2_O without additional tube compensation, as ATC is not routinely available in our ICUs and its use during SBT remains controversial.

Weaning failure was defined as either (1) SBT failure, or (2) successful SBT followed by reintubation or noninvasive ventilation within 48 h after extubation (12). SBT failure was defined by any of the following (13): RR >35 breaths/min; SaO_2_ < 90% despite FiO_2_ ≥ 50%; HR >140 beats/min or ≥20% increase from baseline; systolic blood pressure ≥180 mmHg or ≥20% increase from baseline; or altered mental status, agitation, or anxiety.

Extubation success was defined as maintaining spontaneous breathing without reintubation or noninvasive ventilation for >48 h after extubation (16). Baseline data included age, sex, ICU length of stay, and duration of MV before weaning.

Extubation failure was defined as reintubation or the need for noninvasive ventilation (NIV) within 48 h after extubation. For the purpose of this study, NIV was classified as “rescue NIV” when initiated for signs of acute respiratory distress (respiratory rate >30 breaths/min, SpO_2_ < 90% despite FiO_2_ ≥ 0.5, use of accessory muscles, or hemodynamic instability) after extubation. Prophylactic NIV—defined as NIV planned and initiated immediately after extubation in patients judged at high risk of reintubation without preceding signs of respiratory distress—was not classified as extubation failure in a sensitivity analysis. High-risk criteria for prophylactic NIV included age >65 years, APACHE II >50, obesity (BMI >30 kg/m^2^), or chronic lung disease.

### EIT monitoring and parameter definitions

Regional lung ventilation was monitored continuously using the Infivision 1,900 impedance imaging system (Infivision Medical Technology Co., Ltd., Shenzhen, China). The system was used in conventional single-plane 2D mode with one 16-electrode belt placed circumferentially around the chest at the level of the 4th−5th intercostal space, with the reference line positioned at the mid-axillary line. Electrode contact impedance was maintained below 20 kΩ with balanced inter-electrode impedance. EIT data were continuously acquired at a sampling frequency of 50 Hz, and images were reconstructed using the standardized Graz Consensus Reconstruction algorithm for EIT (GREIT) algorithm (version 2.0) implemented in the Infivision 1,900 software (firmware version 3.2.1). Reconstruction was performed in 2D using the adjacent current injection pattern with a total of 32 × 32 pixels per image. No craniocaudal data were acquired as only a single electrode belt was used. The spatial resolution of the system is approximately 1–2 cm in the imaging plane (14). EIT parameters were recorded and averaged across four predefined phases: 10 min before SBT initiation (T1, Baseline), 0–10 min after SBT initiation (T2, Early SBT), 10–20 min after SBT initiation (T3, Middle SBT), and 20–30 min after SBT initiation (T4, Late SBT).

EIT data were continuously acquired throughout the SBT. For patients who met SBT failure criteria prior to completing the 30-minute trial, the SBT was immediately terminated according to the predefined safety protocol. The exact failure times were recorded: 3 patients at 26 min, 6 at 27 min, 5 at 28 min, 3 at 29 min, and 2 at 30 min. Since no patient failed before 20 min, all 52 patients contributed valid EIT measurements at T1, T2, and T3. For T4 (20–30 min), the calculation method differed based on trial outcomes. For SBT-success patients (*n* = 33, all completed the full 30-minute trial), T4 was calculated as the mean impedance value during the final 10 min of the SBT (20–30 min), using the full available 10-minute data segment consistent with the T2 (0–10 min) and T3 (10–20 min) definitions. For SBT-failure patients (*n* = 19, failed between 26 and 30 min), T4 was defined as the “cut-off” value at failure, calculated as the mean impedance value during the last 60 s before SBT termination. The shortest available averaging window was 60 s (for patients who failed at 26 min), and the longest was 180 s (for patients who failed at 30 min), with a mean averaging window of 108 ± 45 s. Thus T4 was the 10 min mean over 20–30 min in SBT-success patients, and the 60 s mean immediately before termination in SBT-failure patients (range 60–180 s according to failure time; mean 108 ± 45 s). This outcome-dependent difference in averaging windows is acknowledged as a limitation. No imputation, extrapolation, or last-observation-carried-forward methods were used. Valid measurements at each time point are summarized in Supplementary Table S4. To quantify regional ventilation distribution, several established EIT-derived indices were utilized. The ventrodorsal ventilation asymmetry was assessed using the absolute difference in the percentage of ventilation between the ventral (V) and dorsal (D) regions of interest, denoted as |ROI V-D| (17). The COV was employed to represent the geometric center of ventilation distribution along the ventrodorsal axis (ranging from 0% to 100%), with higher values indicating a significant shift in ventilation toward the dorsal (gravity-dependent) lung zones (18). The GI, calculated from pixel-level impedance changes, was used to quantify heterogeneity in ventilation distribution, with higher values indicating greater ventilation mismatch (19).

End-expiratory lung impedance (EELI) was measured as the impedance at the end of expiration during each respiratory cycle, expressed in arbitrary units (AU) as provided directly by the EIT device. Changes in EELI (ΔEELI) were calculated as absolute differences between EELI measurements at different time points:

- ΔEELI (T2-T1) = T2 EELI − T1 EELI
- ΔEELI (T3-T2) = T3 EELI − T2 EELI
- ΔEELI (T4-T3) = T4 EELI − T3 EELI
- ΔEELI (T4-T1) = T4 EELI − T1 EELI

Notably, ΔEELI (T4-T1) = ΔEELI (T2-T1) + ΔEELI (T3-T2) + ΔEELI (T4-T3). Therefore, ΔEELI (T4-T1) represents the cumulative net change in EELI over the entire SBT and is mathematically dependent on the preceding interval changes. The interval changes (T2-T1, T3-T2, T4-T3) are presented for descriptive purposes to illustrate the temporal pattern of EELI changes across different phases of the SBT. The cumulative change ΔEELI (T4-T1) is used as a single summary measure representing the net change over the entire SBT.

### Sample size calculation

Sample size was calculated based on the weaning failure rate observed in our previous study on ultrasound-guided weaning (30%) (20, 21) and supplemented by published data (22), a type I error of 0.05, and a power of 90%, a minimum of 50 patients was required to detect an AUC of 0.80 with a 95% confidence interval width of 0.15. Accounting for a potential 10% dropout rate due to technical issues, we aimed to enroll 55 patients. Ultimately, 52 patients were included in the analysis, meeting the minimum requirement. Sample size calculation was performed using MedCalc® (MedCalc Software Ltd, Ostend, Belgium).

### Statistical analysis

#### Primary longitudinal analysis

The primary analysis was prespecified as the characterization of temporal trajectories of EIT-derived parameters (GI index, COV, |ROI V-D|, and ΔEELI) during the SBT using generalized estimating equations (GEE) with group-by-time interactions. GEE models incorporated fixed effects of outcome group, categorical time (T1–T4), and group-by-time interaction, with individual patients defined as cluster variables. Holm-Bonferroni correction was applied to all pairwise between-group comparisons at each time point. For COV, which exhibited significant baseline (T1) between-group imbalance, baseline-adjusted analyses (ANCOVA and ΔCOV change-score analysis) were performed to eliminate confounding by baseline differences. Interval ΔEELI changes and cumulative ΔEELI (T4–T1) were compared between groups using independent t-tests or Mann–Whitney U tests, with Holm-Bonferroni correction applied across all four comparisons. Only the cumulative ΔEELI (T4–T1) was incorporated into exploratory prediction models as a unified summary measure; interval changes were used solely for descriptive temporal pattern analysis.

#### Secondary exploratory prediction analyses

Secondary analyses examined associations of prespecified EIT parameters with SBT failure (19 events) and extubation failure (9 events). All logistic models coded 1 = failure and 0 = success, so that OR > 1 indicates higher odds of failure. For SBT failure, multivariable models were limited to two prespecified predictors (T4 RSBI and T3 GI; EPV ≥ 9.5). For extubation failure, Firth penalized logistic regression was used only to estimate associations in the dual-predictor model (T4 RSBI + ΔEELI T4–T1). A separately specified unpenalized maximum-likelihood (MLE) model with the same predictors was used for discrimination, calibration, Brier score, and Harrell bootstrap internal validation (1,000 resamples). Apparent AUCs, optimism-corrected AUCs, bootstrap confidence intervals, and prediction-model coefficients correspond to this MLE model and should not be interpreted as Firth estimates. Technical procedures and full outputs are in Supplementary Methods and Supplementary Tables S1–S3 and S8. These prediction analyses are hypothesis-generating.

All statistical tests were two-tailed, and a *P* value <0.05 was defined as statistically significant.

## Results

### Study population and patient flow

A total of 95 patients who were mechanically ventilated for more than 48 h and underwent their first SBT were initially screened for eligibility. Among them, 43 patients were excluded based on the following criteria: tracheostomy (*n* = 15), cardiac pacemaker implantation (*n* = 17), rib fractures (*n* = 2), neuromuscular diseases (*n* = 3), chest skin lesions (*n* = 3), and refusal to participate (*n* = 3).

Consequently, 52 patients were enrolled and included in the final analysis (Figure 1). Based on the SBT outcomes, 33 patients (63.5%) successfully passed the SBT, while 19 patients (36.5%) failed. Among the 33 patients who passed the SBT and proceeded to extubation, 24 (72.7%) were successfully extubated, whereas 9 (27.3%) experienced extubation failure (Figures 2, 3).

**Figure 1:**
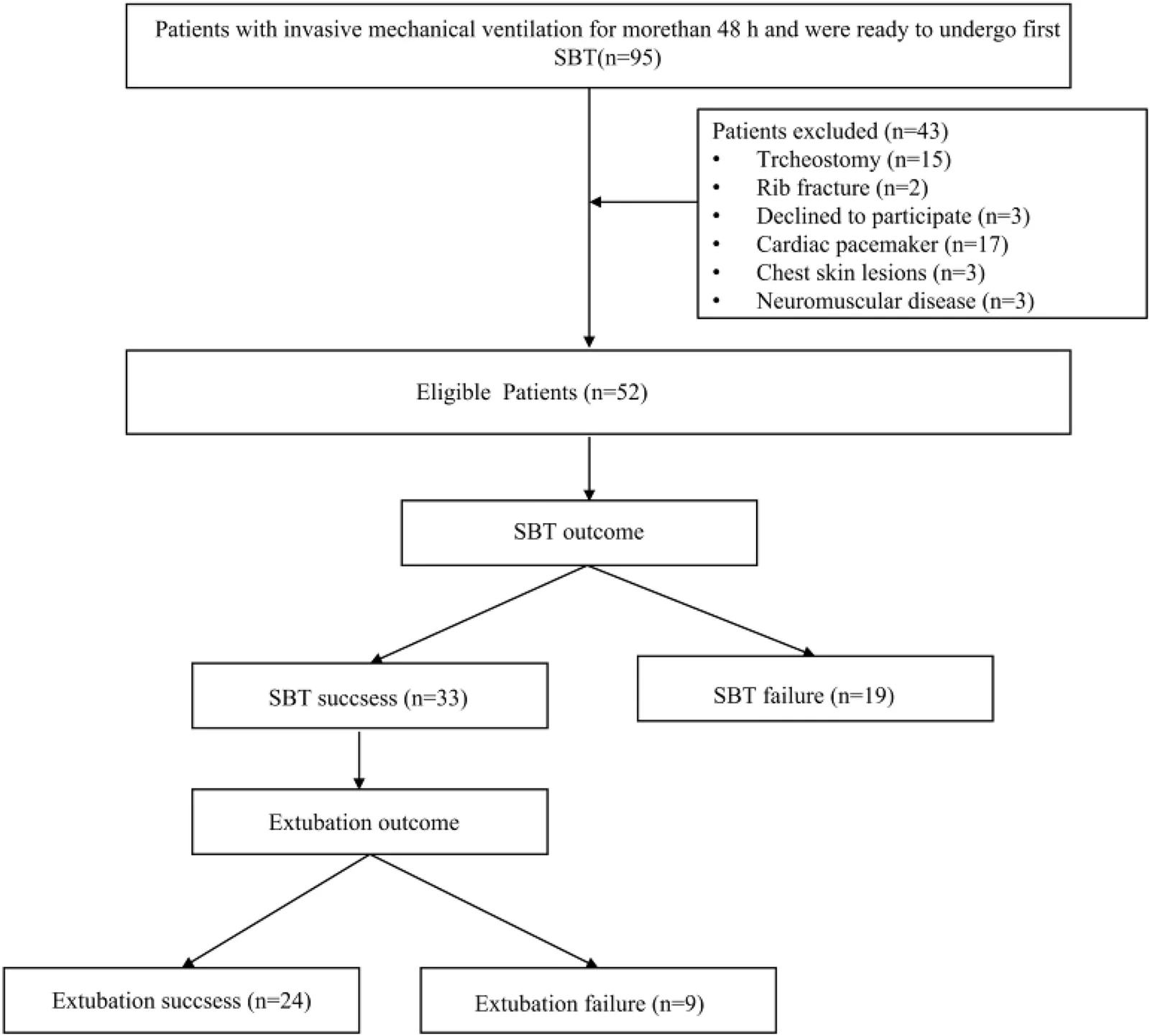
Flowchart of participant screening.

**Figure 2:**
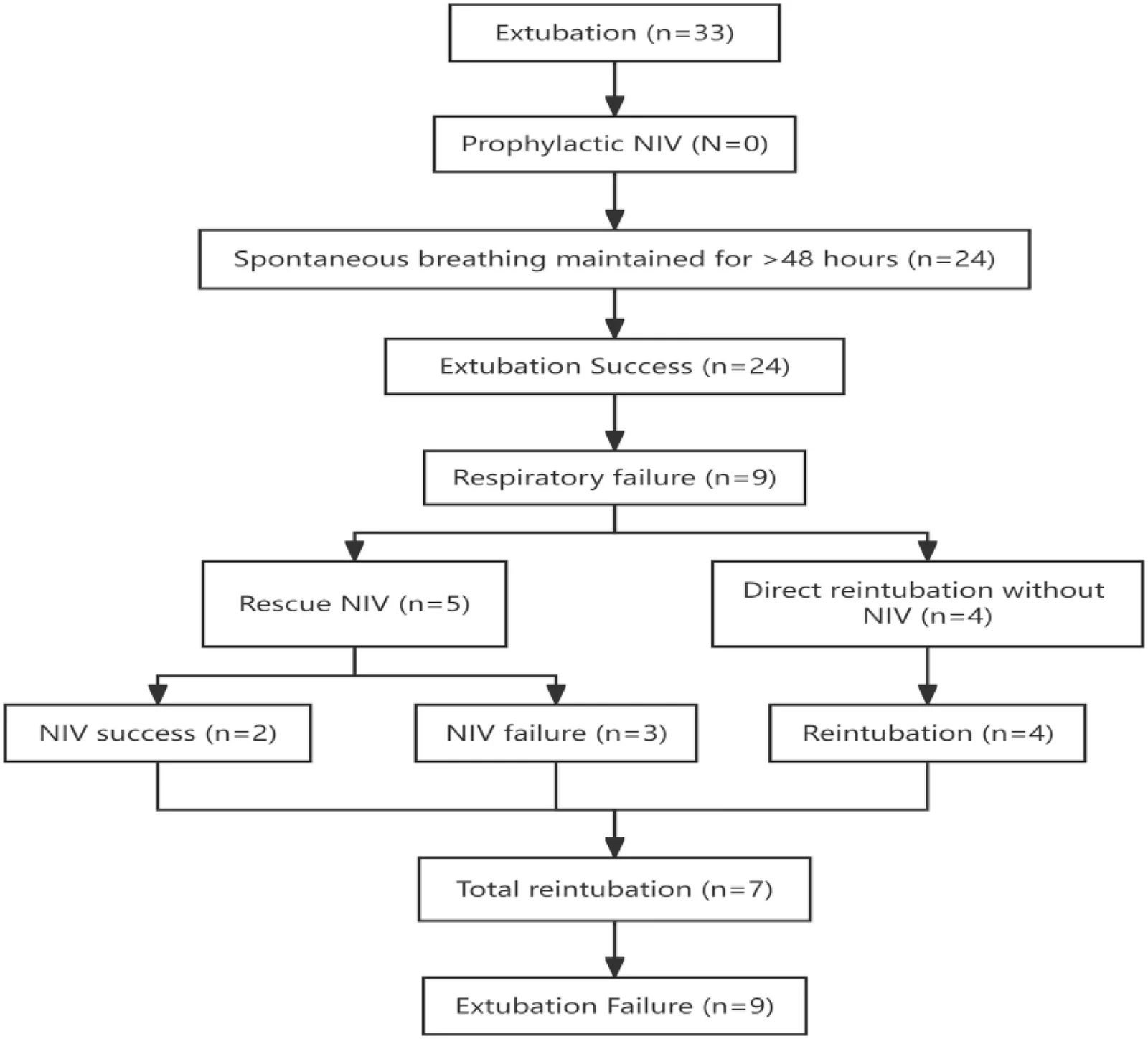
Post-extubation liberation pathway. Following successful SBT (*n* = 33), all patients were extubated within 2 h. No patient received prophylactic NIV immediately after extubation. Among the 33 extubated patients, 24 (72.7%) maintained spontaneous breathing for >48 h and were classified as extubation success. Nine patients (27.3%) developed post-extubation respiratory failure: 5 received rescue NIV (of whom 2 successfully avoided reintubation, while 3 ultimately required reintubation), and 4 underwent direct reintubation without prior NIV Overall, 7 of the 9 extubation failure patients ultimately required reintubation (3 after failed NIV trial, 4 directly). Only 2 patients who received rescue NIV successfully avoided reintubation. NIV, noninvasive ventilation; SBT, spontaneous breathing trial; h, hours.

**Figure 3:**
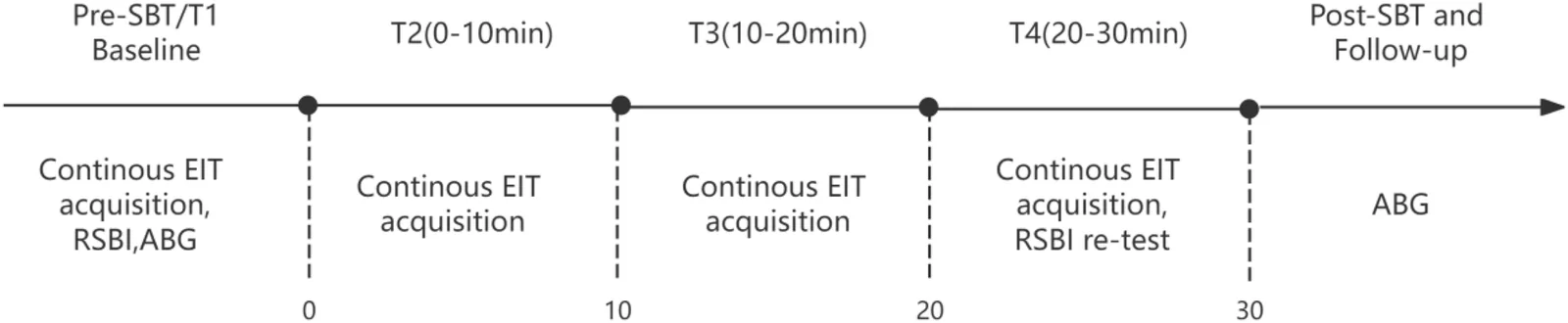
Timeline of SBT monitoring and data collection. T1 represents baseline measurement 10 min before SBT initiation; SBT commenced at 0 min with ventilator settings of PSV 5 cmH2O and PEEP 0 cmH_2_O; Continuous electrical impedance tomography (EIT) data were recorded throughout T2 (0–10 min), T3 (10–20 min), and T4 (20–30 min); Rapid shallow breathing index (RSBI) and arterial blood gas (ABG) samples were collected at T1 and T4. Nineteen patients met SBT failure criteria between 26 and 30 min of the trial; Patients who passed SBT received extubation assessment within 2 h, with clinicians blinded to all EIT-derived parameters; Post-extubation follow-up was performed within 48 h to monitor reintubation or non-invasive ventilation requirements.

### Baseline characteristics

Fifty-two patients were included in this study. Their baseline characteristics are detailed in Table 1. The mean age was 68.48 ± 18.61 years, with 31 (59.62%) males. The mean APACHE II score was 22.27 ± 6.46, and the median SOFA score was 9 [6–10].

**Table 1:** Baseline characteristics of study patient.

Variables	All patients (*N* = 52)	SBT success (*n* = 33)	SBT failure (*n* = 19)	*P* (SBT)	All patients (*n* = 33)	Extubation success (*n* = 24)	Extubation failure (*n* = 9)	*P* (Extubation)
Demographics								
Age (years), mean ± SD	68.48 ± 18.61	68.18 ± 17.45	69.00 ± 20.98	0.88	68.18 ± 17.45	67.50 ± 19.34	70 ± 11.69	0.72
Male, *n* (%)	31 (59.62%)	19 (57.58%)	12 (63.16%)	0.696	19 (57.58%)	14 (58.33%)	5 (55.56%)	0.921
Height (cm), median [IQR]	168 [157–172]	168 [157–171]	168 [157–172]	0.761	168 [157–171]	168 [156–171]	168 [158–172]	0.627
Weight (kg), mean ± SD	67.77 ± 9.38	66.76 ± 10.27	69.53 ± 7.53	0.31	66.76 ± 10.27	66.71 ± 10.82	66.89 ± 9.23	0.965
Body mass index (kg/m^2^), mean ± SD	24.79 ± 3.95	24.48 ± 3.67	25.29 ± 2.10	0.523	24.71 ± 4.34	24.79 ± 4.55	24.48 ± 3.95	0.858
Severity of illness								
APACHE II score, mean ± SD	22.27 ± 6.46	19.94 ± 5.32	26.32 ± 6.09	<0.001	19.94 ± 5.32	20.75 ± 5.88	20.11 ± 4.01	0.096
SOFA score, median [IQR]	9 [6–10]	9 [5.5–10]	9 [6–11]	0.334	9 [5.5–10]	8.5 [5.5–10.0]	9 [7–9]	0.890
Comorbidities, *n* (%)								
Hypertension	17 (32.69%)	10 (30.30%)	7 (36.84%)	0.643	10 (30.30%)	8 (33.33%)	2 (22.22%)	0.512
Diabetes, *n* (%)	16 (30.77%)	10 (30.30%)	6 (31.58%)	0.935	10 (30.30%)	8 (33.33%)	2 (22.22%)	0.512
Chronic lung disease/COPD	8 (15.38%)	6 (18.18%)	2 (10.53%)	0.496	6 (18.18%)	3 (12.50%)	3 (33.33%)	0.193
Congestive heart failure	6 (11.54%)	4 (12.12%)	2 (10.53%)	0.835	4 (12.12%)	2 (8.33%)	2 (22.22%)	0.281
Renal failure	5 (9.62%)	3 (9.09%)	2 (10.53%)	0.887	3 (9.1%)	3 (12.5%)	0 (0%)	**0**.046*
Reason for intubation, *n* (%)								
Respiratory failure	10 (19.23%)	7 (21.21%)	3 (15.79%)	0.696	7 (21.21%)	6 (25.00%)	1 (11.11%)	0.394
Neurological conditions	9 (17.31%)	6 (18.18%)	3 (15.79%)	0.796	6 (18.18%)	4 (16.67%)	2 (22.22%)	0.727
Cardiac failure/Pulmonary edema	7 (13.46%)	4 (12.12%)	3 (15.79%)	0.719	4 (12.12%)	3 (12.50%)	0 (0%)	0.291
Post-surgery	9 (17.31%)	5 (15.15%)	4 (21.05%)	0.593	5 (15.15%)	1 (4.17%)	4 (44.44%)	**0**.002*
Septic shock	7 (13.46%)	3 (9.09%)	4 (21.05%)	0.229	3 (9.09%)	3 (12.50%)	0 (0%)	0.291
Others	10 (19.23%)	8 (24.24%)	2 (10.53%)	0.237	8 (24.24%)	6 (25.00%)	2 (22.22%)	0.858
Clinical status before SBT								
Duration of MV before SBT (days), median [IQR]	5 [3–6]	5 [3–6.5]	4 [3–6]	0.248	5 [3–6.5]	4.5 [3–6]	5 [2–7]	0.890
Use of diuretics, *n* (%)	35 (67.31%)	22 (66.67%)	13 (68.42%)	0.898	22 (66.67%)	14 (58.33%)	8 (88.89%)	0.193
Ventilatory parameters								
Pressure support level (cmH_2_O), median [IQR]	9 [9–10]	9 [9–10]	9 [9–10]	0.828	9 [9–10]	9 [9–10]	10 [8–10]	0.736
PEEP (cmH_2_O), median [IQR]	4 [4–5]	4 [4–5]	4 [3–5]	0.152	4 [4–5]	4.5 [4–5]	4 [4–5]	0.414
FiO_2_ (%), median [IQR]	0.4 [0.35–0.4]	0.4 [0.35–0.4]	0.4 [0.35–0.4]	0.295	0.4 [0.35–0.4]	0.4 [0.35–0.40]	0.4 [0.35–0.40]	0.890
Tidal volume (VT) before SBT (L), median [IQR]	0.45 [0.41–0.49]	0.44 [0.41–0.51]	0.46 [0.41–0.49]	0.499	0.44 [0.41–0.51]	0.45 [0.42–0.51]	0.41 [0.41–0.45]	0.111
Respiratory rate (RR) before SBT (bpm), median [IQR]	15 [13–18]	15 [13–17]	16 [15–19]	0.117	15 [13–17]	15 [13–15]	15 [14–20]	0.370
RSBI before SBT (breaths/min/L), median [IQR]	33.71 [27.11–38.99]	33.33 [26.32–37.5]	34.15 [32.63– 40.16]	0.318	33.33 [26.32–37.5]	32.26 [26.78–36.72]	36.59 [26.32–48.78]	0.107
Tidal volume (VT) after SBT (L), median [IQR]	0.41 [0.36–0.45]	0.42 [0.38–0.45]	0.36 [0.30–0.41]	**0**.007*	0.42 [0.38–0.45]	0.43 [0.39–0.46]	0.41 [0.38–0.45]	0.593
Respiratory rate (RR) after SBT (bpm), median [IQR]	16.5 [15–18.75]	15 [13–17]	21 [18–23.5]	**<0**.001*	15 [13–17]	15 [12.5–17]	15 [15–16]	0.394
RSBI after SBT (breaths/min/L), mean ± SD	45.17 ± 20.66	35.00 ± 7.55	62.83 ± 24.27	**<0**.001*	35.00 ± 7.55	34.24 ± 8.28	37.04 ± 4.92	0.350
Arterial blood gas								
PaO_2_/FiO_2_ before SBT (mmHg), median [IQR]	384 [313.95–464.5]	401 [318.8–500.3]	304.6 [302–471.4]	0.157	401 [318.8–500.3]	414.90 [344.18–538.68]	380.9 [321.4–399.8]	0.131
PaCO_2_ before SBT (mmHg), median [IQR]	37.75 [34.65–41.45]	36.8 [33.7–41.1]	38.8 [35.1–42.5]	0.151	36.8 [33.7–41.1]	37.3 [34–41.4]	36.8 [33.7–38.2]	0.540
pH before SBT, mean ± SD	7.42 ± 0.04	7.41 ± 0.04	7.42 ± 0.04	0.455	7.41 ± 0.04	7.41 ± 0.04	7.43 ± 0.04	0.201
PaO_2_ before SBT (mmHg), mean ± SD	151.99 ± 45.98	160.62 ± 52.85	136.99 ± 25.48	0.074	160.62 ± 52.85	160.41 ± 57.15	137.2 ± 30.57	0.072
FiO_2_ before SBT (%), median [IQR]	0.4 [0.35–0.4]	0.4 [0.35–0.4]	0.4 [0.35–0.4]	0.295	0.4 [0.35–0.4]	0.4 [0.35–0.4]	0.4 [0.35–0.4]	0.890
PaO_2_/FiO_2_ after SBT (mmHg), median [IQR]	352.19 [292.93–399.63]	375.3 [321.5–444]	287.3 [235.05–346.55]	**<0**.001*	375.3 [321.5–444]	398.35 [368.45–512.35]	321.5 [308.75–339.00]	**0**.004*
PaCO_2_ after SBT (mmHg), mean ± SD	41.02 ± 6.02	40.06 ± 5.47	42.69 ± 6.71	0.131	40.06 ± 5.47	39.70 ± 5.23	41.03 ± 6.29	0.540
pH after SBT, median [IQR]	7.43 [7.40–7.46]	7.43 [7.41–7.46]	7.43 [7.40–7.46]	0.607	7.43 [7.41–7.46]	7.445 [7.42–7.47]	7.4 [7.42–7.44]	0.223
PaO_2_ after SBT (mmHg), median [IQR]	142.35[119.98–168.33]	150.1 [128.6–185.5]	124.4 [104.5–144.65]	**0**.001*	150.1 [128.6–185.5]	163.05[141.1–195.9]	128.6[119.0–144.7]	0.015
FiO_2_ after SBT (%), median [IQR]	0.4 [0.4–0.45]	0.4 [0.35–0.45]	0.45 [0.4–0.45]	0.089	0.4 [0.35–0.45]	0.4 [0.4–0.45]	0.4 [0.4–0.5]	1.000
Clinical Outcomes								
ICU length of stay (days), median [IQR]	18.5 [10.25–25]	14 [10–23]	27 [15–28]	**0**.009*	14 [10–23]	17 [10.5–23]	10 [8–17]	0.134
ICU Mortality, *n* (%)	2 (3.85%)	1 (3.03%)	1 (5.26%)	0.694	1 (3.03%)	1 (4.17%)	0 (0%)	0.858

* Indicates statistical significance (*P* < 0.05).

Continuous variables are presented as mean ± standard deviation (SD) or median [interquartile range (IQR)] as appropriate.

Categorical variables are presented as a number (%).

The counts for renal failure in the extubation analysis have been re-verified from the source data; the corrected numbers are 3 (12.5%) in the extubation success group and 0 (0%) in the extubation failure group, with a total of 3 (9.1%) among all extubated patients.

For variables that were normally distributed and compared using t-tests, the summary statistics are presented as median [IQR] in this table solely to maintain a uniform format across all continuous variables. The statistical inferences (*P*-values) remain based on t-tests, as these variables met the normality assumption.

APACHE II, acute physiology and chronic health evaluation II; SOFA: sequential organ failure assessment; COPD, chronic obstructive pulmonary disease; MV, mechanical ventilation; SBT, spontaneous breathing trial; PEEP, positive end-expiratory pressure; FiO, fraction of inspired oxygen; VT, tidal volume; RR, respiratory rate; RSBI, rapid shallow breathing index; PaO_2_, partial pressure of oxygen in arterial blood; PaCO_2_, partial pressure of carbon dioxide in arterial blood; pH, potential of hydrogen; ICU, intensive care unit.

SBT failure (*n* = 19) was associated with a higher APACHE II score (26.32 ± 6.09 vs. 19.94 ± 5.32, *p* < 0.001) and longer ICU length of stay [27 [15–28] days vs. 14 [10–23] days, *p* = 0.009] compared to SBT success (*n* = 33). Post-SBT, the failure group exhibited significantly lower VT [0.36 [0.30–0.41] L vs. 0.42 [0.38–0.45] L, *p* = 0.007], higher RR (21 [18–23.5] bpm vs. 15 [13–17] bpm, *p* < 0.001), and higher RSBI (62.83 ± 24.27 vs. 35.00 ± 7.55, *p* < 0.001). PaO_2_/FiO_2_ (287.3 [235.05–346.55] mmHg vs. 375.3 [321.5–444] mmHg, *p* < 0.001) and PaO_2_ (124.4 [104.5–144.65] mmHg vs. 150.1 [128.6–185.5] mmHg, *p* = 0.001) were also significantly lower in the failure group.

Among SBT-successful patients (*n* = 33), 24 achieved extubation success, while 9 failed. Extubation failure was associated with a higher incidence of post-surgery as the reason for intubation (44.44% vs. 4.17%, *p* = 0.002), whereas renal failure was more prevalent in the success group (12.5% vs. 0%, *p* = 0.046). Post-SBT, the extubation failure group showed lower PaO_2_/FiO_2_ (321.5 [308.75–339.00] mmHg vs. 398.35 [368.45–512.35] mmHg, *p* = 0.004) and PaO_2_ (163.05[141.1–195.9] mmHg vs. 128.6 [119.0–144.7] mmHg, *p* = 0.015).

### Patients contributing to EIT measurements at each time point

Among the 52 enrolled patients, all completed the baseline measurement (T1) and T2 (0–10 min) measurements. At T3 (10–20 min), all 52 patients were still undergoing the SBT and provided valid EIT data. At T4 (20–30 min), although 19 patients met SBT failure criteria before completing the full 30-minute trial (failure times: 26 min: *n* = 3, 27 min: *n* = 6, 28 min: *n* = 5, 29 min: *n* = 3, 30 min: *n* = 2), all patients had a valid T4 value based on the last measurement before termination (cut-off value). Thus, all 52 patients contributed valid EIT data at all four time points. A detailed summary is provided in Supplementary Table S4. No data were imputed.

### Dynamic EIT parameter analysis

Generalized estimating equations (GEE) revealed significant group-by-time interaction effects for the GI index (*p* < 0.001), |ROI V-D| (*p* = 0.003), and COV (*p* = 0.001), indicating distinct temporal trajectories of ventilation distribution between the SBT success and failure groups (Table 2). After Holm-Bonferroni correction for multiple comparisons, the failure group showed significantly higher GI index at T3 (adjusted *p* = 0.018) and T4 (adjusted *p* < 0.001), and higher |ROI V-D| at T3 (adjusted *p* = 0.021), compared to the success group. In the failure group, the GI index progressively increased from T1 to T4 (42.37 ± 1.18 to 50.63 ± 1.17), whereas it remained stable in the success group. For |ROI V-D|, the failure group exhibited a pronounced spike at T3 (20.37 ± 2.96), significantly higher than that in the success group (11.24 ± 1.29, adjusted *p* = 0.021), reflecting a transient but severe ventrodorsal ventilation imbalance.

**Table 2:** Dynamic evolution of EIT parameters during the spontaneous breathing trial (SBT).

Parameter	Group	T1	T2	T3	T4	P_interaction
GI Index	Success (*n* = 33)	44.94 ± 0.67	46.61 ± 0.64	44.64 ± 0.61	45.97 ± 0.59	<0.001
	Failure (*n* = 19)	42.37 ± 1.18	48.32 ± 1.39	48.37 ± 1.22	50.63 ± 1.17	
	P_diff	0.058	0.263	0.006*	<0.001*	
	P_adj (Holm)	0.116	0.263	0.018	<0.001	
|ROI V-D|	Success (*n* = 33)	13.82 ± 1.02	11.70 ± 0.86	11.24 ± 1.29	14.55 ± 1.50	0.003
	Failure (*n* = 19)	12.32 ± 1.26	14.26 ± 2.80	20.37 ± 2.96	18.05 ± 2.57	
	P_diff	0.355	0.381	0.005*	0.239	
	P_adj (Holm)	0.355	0.381	0.021	0.239	
COV (%)	Success (*n* = 33)	46.97 ± 0.92	47.85 ± 0.93	46.15 ± 0.95	45.33 ± 0.87	0.001
	Failure (*n* = 19)	42.42 ± 1.05	48.74 ± 1.21	41.79 ± 1.07	41.74 ± 1.11	
	P_diff	0.001*	0.561	0.002*	0.011*	
	P_adj (Holm)	0.003	0.561	0.016	0.035	

* Indicates statistical significance (*p* < 0.05).

Data are presented as predicted marginal means ± standard error (SE) derived from Generalized Estimating Equations (GEE) models.

P_interaction represents the significance of the Group × Time interaction effect.

P_diff represents the significance of the difference between the Success and Failure groups at each specific time point.

The *P*-values after Holm–Bonferroni correction are presented in the main text.

GI, global inhomogeneity; ROI V-D, absolute difference in ventilation between ventral and dorsal regions of interest; COV, center of ventilation.

Regarding COV, the failure group showed an abrupt dorsal shift at T2 followed by a subsequent ventral decline, whereas the success group maintained a more stable distribution with consistently more dorsal orientation at T1, T3, and T4. After Holm-Bonferroni correction, the failure group showed significantly lower COV at T1 (adjusted *p* = 0.003), T3 (adjusted *p* = 0.016), and T4 (adjusted *p* = 0.035) compared to the success group.

To address the potential confounding effect of baseline differences in COV, we performed a Supplementary change-score analysis (ΔCOV = COV at Tx−COV at T1) as prespecified in the statistical analysis. The failure group showed a significant ventral shift from T1 to T3 (mean ΔCOV: −0.63%, 95% CI: −1.12 to −0.14, *p* = 0.014) and T4 (mean ΔCOV: −0.68%, 95% CI: −1.21 to −0.15, *p* = 0.013), indicating that the ventral redistribution of ventilation was progressive and sustained, whereas the success group showed stable values with minimal change from baseline (T3: −0.82%, *p* = 0.312; T4: −1.64%, *p* = 0.108). Detailed results are presented in Supplementary Table S5. These findings confirm that the temporal trajectory differences in COV were not attributable solely to baseline differences, and that the observed ventral shift in the failure group represents a true dynamic change during the SBT.

Descriptive interval analysis of end-expiratory lung impedance changes revealed distinct temporal EELI trajectories between patients with successful and failed SBT. The SBT success group maintained stable EELI throughout the entire trial period, whereas the SBT failure group exhibited progressive EELI decline. Specifically, the failure group demonstrated significantly greater ΔEELI reduction during the late SBT phase (T4–T3, *p* < 0.001) and across the cumulative SBT period (T4–T1, *p* < 0.01), consistent with an associated pattern of progressive reduction in end-expiratory lung impedance. Representative EIT images further illustrated these divergent physiological patterns (Figure 4): patients with successful SBT showed improved ventilation homogeneity and stable or elevated EELI, with sustained positive ΔEELI and homogeneous blue impedance distribution throughout the trial. In contrast, patients with SBT failure exhibited progressive ventilation inhomogeneity, elevated GI index, and persistent EELI reduction, characterized by expanding low-impedance orange regions and consistently negative ΔEELI. Detailed temporal trends of regional ventilation parameters during SBT are presented in Supplementary Figures S4–S6 (Figure 5).

**Figure 4:**
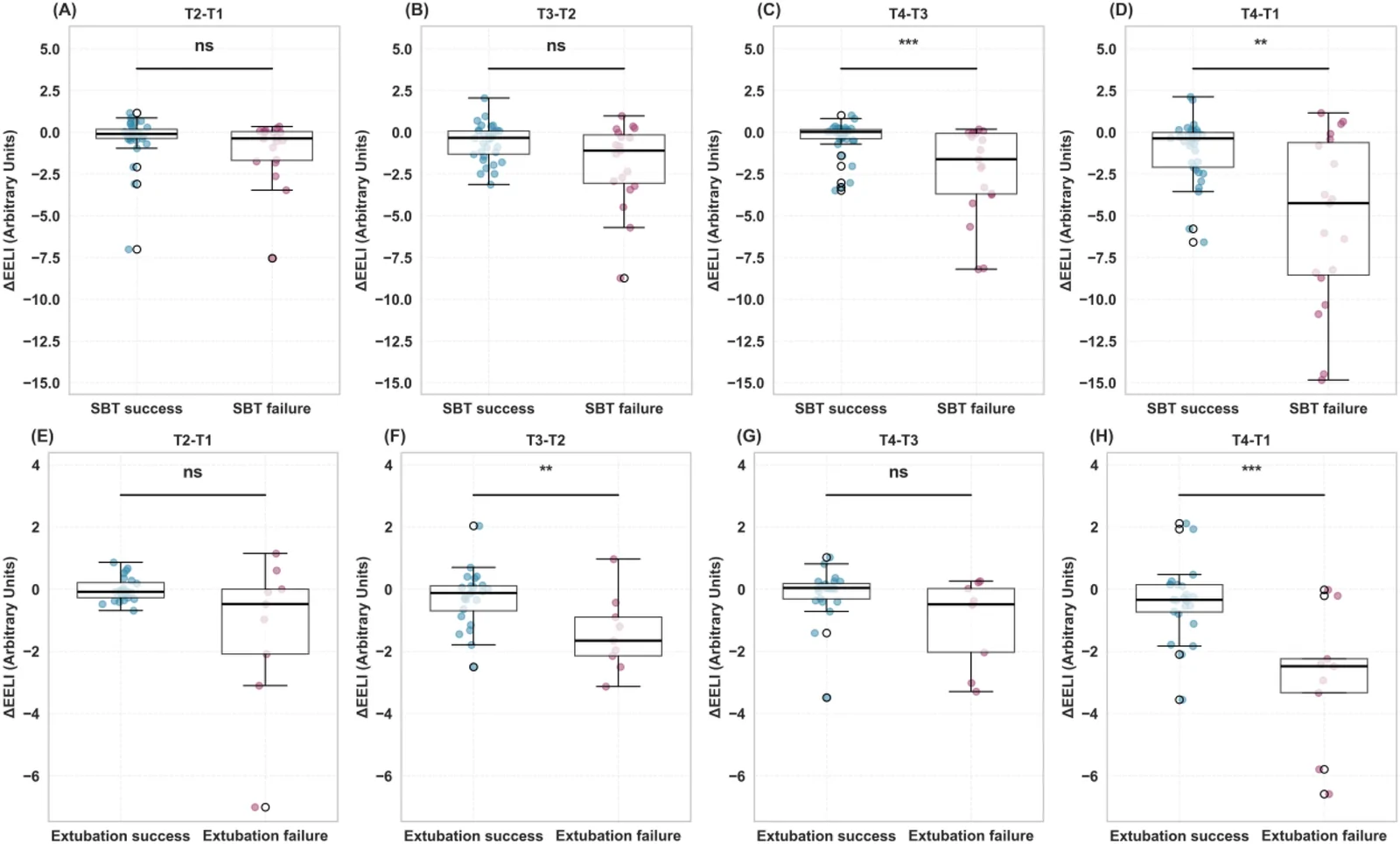
Changes in end-expiratory lung impedance (ΔEELI) during the spontaneous breathing trial (SBT) for SBT and extubation outcomes. Descriptive comparison of ΔEELI between groups across different time intervals during the spontaneous breathing trial (SBT). ΔEELI values were calculated as absolute differences between EELI measurements at different time points: ΔEELI (T2-T1) = T2 EELI − T1 EELI; ΔEELI (T3-T2) = T3 EELI − T2 EELI; ΔEELI (T4-T3) = T4 EELI − T3 EELI; ΔEELI (T4-T1) = T4 EELI − T1 EELI. The upper row **(Panels A–D)** illustrates ΔEELI between the SBT success group (blue) and the SBT failure group (red) for intervals T2-T1, T3-T2, T4-T3, and T4-T1, respectively. The lower row **(Panels E–H)** presents ΔEELI between the extubation success group (blue) and the extubation failure group (red) for the same time intervals. These interval changes are presented for descriptive purposes to illustrate the temporal pattern of EELI changes across different phases of the SBT. Only the cumulative change ΔEELI (T4-T1) was used as a single summary measure in predictive models. Box plots represent the median and interquartile range (IQR), and individual data points are overlaid. Statistical significance is indicated as: ns, not significant; **, *p* < 0.01; ***, *p* < 0.001.

**Figure 5:**
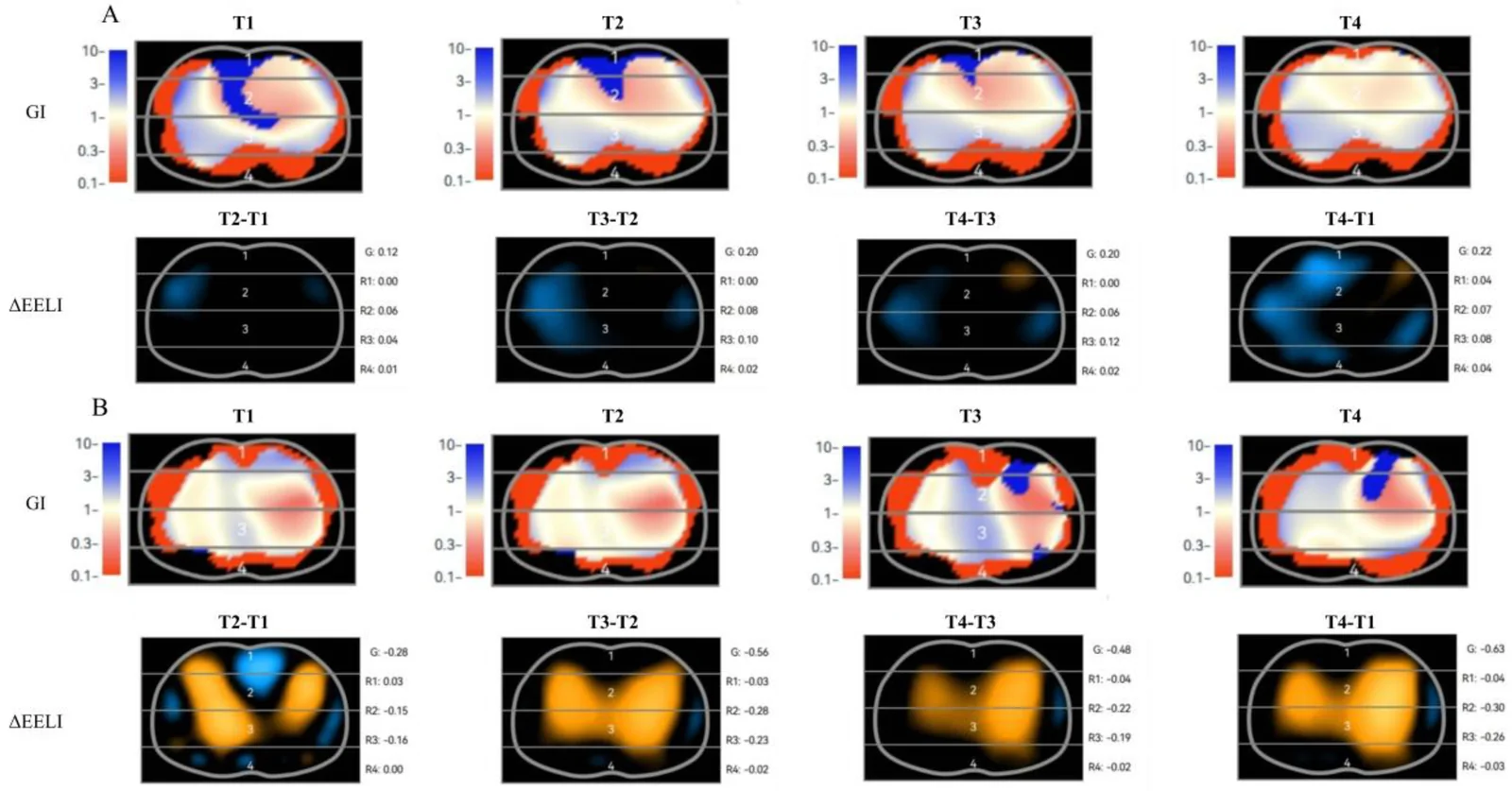
Representative EIT images of two patients during a spontaneous breathing trial (SBT). The color scale indicates changes in impedance relative to T1 (baseline), where blue represents an increase in impedance (suggesting lung volume increase or recruitment) and orange represents a decrease in impedance (suggesting lung volume loss or de-recruitment). The upper row (Patient A) shows a patient with improved ventilation homogeneity and stable or increased end-expiratory lung impedance (EELI), characterized by predominant blue regions and positive ΔEELI throughout the SBT, indicating effective maintenance of lung aeration. The lower row (Patient B) shows a patient with progressive ventilation deterioration, characterized by worsening GI index and a decline in EELI, with expanding orange regions and consistently negative ΔEELI, reflecting progressive lung de-recruitment. T1: 10 min before SBT; T2: 0–10 min into SBT; T3: 10–20 min into SBT; T4: 20–30 min into SBT.

With regard to extubation outcomes, ΔEELI trajectories also differed significantly between extubation success and failure groups. Patients who developed post-extubation respiratory failure presented a significantly greater magnitude of EELI decline during the mid-SBT phase (T3-T2, *p* < 0.01) and over the full SBT duration (T4–T1, *p* < 0.001) compared with patients with successful extubation (LE–H). Notably, conventional regional ventilation parameters, including GI index, COV, and |ROI V-D|, showed no significant between-group differences at any SBT time point for extubation outcomes (all *p* > 0.05; Supplementary Figures S7–S9), indicating that dynamic lung volume derecruitment, rather than altered ventilation distribution, appears to be the predominant associated factor in post-extubation failure.

### Exploratory associations with SBT failure and extubation failure

Exploratory discrimination is summarized in Table 3 and Figure 6. Full univariable and multivariable results at all time points, bootstrap internal validation, incremental value over RSBI, calibration, the T2 landmark analysis, and the reintubation-only sensitivity analysis are reported in Supplementary Tables S1–S8 and Figures S1–S3.

**Table 3:** Logistic regression and ROC analysis of EIT parameters and RSBI for association with SBT failure and extubation failure.

Outcome & variables	EPV	Univariate OR (95% CI)	*P*	Adjusted OR (95% CI)	*P*	AUC (95% CI)	Cut-off
I. Weaning Outcome (SBT Failure)							
T4 RSBI	—	1.17 (1.05–1.26)	0.002	1.12 (1.02–1.24)	0.019	0.874 (0.748–0.979)	55.6
T3 GI Index	—	1.26 (1.06–1.50)	0.010	1.73 (1.15–2.59)	0.008	0.813 (0.644–0.944)	48.0
T3 COV (%)	—	0.85 (0.75–0.96)	0.011	0.92 (0.73–1.16)	0.469	0.714 (0.558–0.852)	39.0
ΔEELI (T4–T3)	—	0.54 (0.35–0.83)	0.005	0.37 (0.15–0.92)	0.032	0.778 (0.634–0.895)	−0.03
Combined (T4 RSBI + T3 GI)	9.5	—	—	—	—	0.923 (0.829–0.986)	—
II. Extubation Outcome (Extubation Failure)							
T4 RSBI	—	1.05 (0.95–1.16)	0.356	1.08 (0.95–1.30)	0.312	0.567 (0.356–0.765)	61.2
ΔEELI (T4–T1)	—	0.35 (0.16–0.77)	0.009*	0.42 (0.18–0.74)	0.012	0.833 (0.629–1.000)	−2.24
ΔEELI (T3-T2)	—	0.33 (0.13–0.81)	0.016*	—	—	0.799 (0.556–0.961)	−0.43
Combined (T4 RSBI + ΔEELI T4–T1)	3.0	—	—	—	—	0.875 (0.688–0.988)	

* Indicates statistical significance (*P* < 0.05).

EPV: Events per variable. For SBT analysis: 19 events, multivariable models limited to ≤2 predictors (EPV ≥ 9.5). For extubation analysis: 9 events, models are exploratory; EPV for combined model = 9/3 = 3.0.

Adjusted ORs for the extubation dual-predictor model (T4 RSBI + ΔEELI T4–T1) are Firth penalized estimates with profile-likelihood 95% CIs (EPV = 3.0); see Supplementary Table S8. Adjusted ORs were not calculated for other extubation variables because of the limited number of events. SBT adjusted ORs are from unpenalized MLE multivariable logistic regression. Apparent AUCs throughout Table 3 are from MLE prediction models, not Firth.

Bootstrap AUC: 95% bootstrap confidence intervals (2,000 resamples) reported for extubation analysis to account for optimism.

Combined Models: AUC values are derived from predicted probabilities of logistic regression models. These are exploratory and require external validation.

ΔEELI values are expressed in arbitrary units (AU). Lower (more negative) ΔEELI indicates greater loss of end-expiratory lung impedance. ΔEELI (T4-T1) represents the cumulative net change over the entire SBT and is mathematically dependent on the preceding interval changes [ΔEELI (T4–T1) = ΔEELI (T2-T1) + ΔEELI (T3-T2) + ΔEELI (T4-T3)]. This measure is used as a single cumulative summary predictor, not as a repeated or interval-based measure.

T4 RSBI denotes RSBI measured at 20–30 min after SBT initiation (or at the time of SBT termination for patients who failed before 30 min).

Variable selection was based on physiological plausibility; no stepwise or automated selection (*P* < 0.10 threshold) was used for any model.

Outcome coding for all logistic models: 1 = failure, 0 = success. OR > 1 indicates increased odds of SBT failure or extubation failure; OR < 1 indicates decreased odds of failure.

Sample sizes: SBT analysis: 52 patients total (33 success, 19 failure). Extubation analysis: 33 patients total (24 success, 9 failure).

Diagnostic performance at the Youden threshold of the MLE combined models (Supplementary Table S1): SBT failure, sensitivity 0.737 (bootstrap 95% CI: 0.538–0.957) and specificity 0.970 (0.839–1.000); extubation failure, sensitivity 0.778 (0.249–1.000) and specificity 0.917 (0.800–1.000).

RSBI, rapid shallow breathing index; EIT, electrical impedance tomography; SBT, spontaneous breathing trial; GI, global inhomogeneity index; COV, center of ventilation; EELI, end-expiratory lung impedance; ΔEELI, change in end-expiratory lung impedance; AUC, area under the receiver operating characteristic curve; OR, odds ratio; CI, confidence interval; EPV, events per variable.

**Figure 6:**
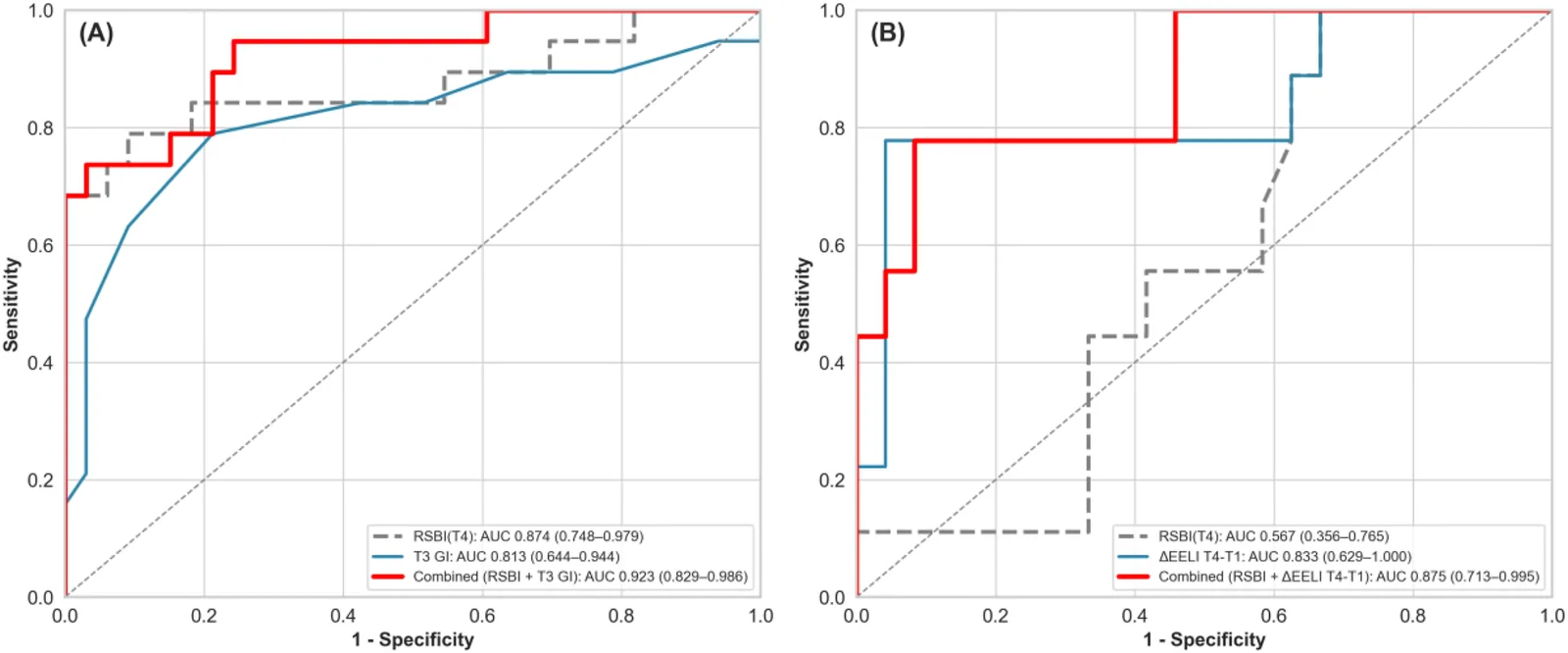
Receiver operating characteristic (ROC) curves of EIT parameters for their association with clinical outcomes. **(A)** ROC curves for T4 RSBI, T3 GI index, and the combined model (T4 RSBI + T3 GI index) in predicting SBT failure. **(B)** ROC curves for T4 RSBI, ΔEELI (T4-T1), and the combined model [T4 RSBI + ΔEELI (T4-T1)] in predicting extubation failure. In this exploratory analysis, the combined MLE model for SBT failure **(A)** had the highest apparent AUC (0.923). For extubation failure **(B)**, ΔEELI (T4-T1) was the strongest single marker. These estimates require external validation. AUC, area under the curve; GI, global inhomogeneity; ΔEELI, change in end-expiratory lung impedance; RSBI, rapid shallow breathing index; SBT, spontaneous breathing trial.

For SBT failure, T4 RSBI (AUC 0.874, 95% CI 0.748–0.979) and T3 GI (AUC 0.813, 95% CI 0.644–0.944) were the strongest single markers. The combined MLE model (T4 RSBI + T3 GI) had an apparent AUC of 0.923 (95% CI 0.829–0.986; optimism-corrected AUC 0.905) and improved model fit over RSBI alone (likelihood-ratio *P* = 0.015; Supplementary Table S3).

For extubation failure (9 events), ΔEELI (T4–T1) had the strongest unadjusted association (AUC 0.833, 95% CI 0.629–1.000). The combined MLE model (T4 RSBI + ΔEELI T4–T1) had an apparent AUC of 0.875 (95% CI 0.688–0.988) and is exploratory. Firth association estimates for the same dual-predictor model and the reintubation-only analysis (7/33) are in Supplementary Table S8.

### Extubation outcome details

Among the 33 patients who underwent extubation, 9 (27.3%) experienced extubation failure according to the primary composite definition (reintubation or rescue NIV). The post-extubation liberation pathway is illustrated in Figure 2, and detailed characteristics of the 9 extubation failure patients are summarized in Table 4.

**Table 4:** Detailed characteristics of extubation failure events (*n* = 9).

Patient	Post-extubation presentation	Time to event	Rescue NIV attempted	NIV success	Final outcome	Cause of reintubation
1	Respiratory distress	18 h	Yes	No	Reintubation at 28 h	Upper airway obstruction
2	Respiratory distress	12 h	Yes	No	Reintubation at 36 h	Respiratory failure/atelectasis
3	Respiratory distress	30 h	Yes	No	Reintubation at 44 h	Cardiovascular instability
4	Respiratory distress	14 h	Yes	Yes	Avoided reintubation	N/A
5	Respiratory distress	22 h	Yes	Yes	Avoided reintubation	N/A
6	Direct respiratory failure	6 h	No	N/A	Reintubation	Respiratory failure/atelectasis
7	Direct respiratory failure	14 h	No	N/A	Reintubation	Cardiovascular instability
8	Direct respiratory failure	22 h	No	N/A	Reintubation	Upper airway obstruction
9	Direct respiratory failure	32 h	No	N/A	Reintubation	Respiratory failure/atelectasis

NIV, noninvasive ventilation; h, hours post-extubation. Rescue NIV was initiated for signs of respiratory distress (respiratory rate >30 breaths/min, SpO_2_ < 90% despite FiO_2_ ≥ 0.5, use of accessory muscles, or hemodynamic instability). Reintubation causes were classified as: upper airway obstruction/secretions (*n* = 2), respiratory failure/atelectasis (*n* = 4), and cardiovascular instability (*n* = 3). Among the 5 patients who received rescue NIV, 3 (60%) ultimately required reintubation, while 2 (40%) successfully avoided reintubation.

No patient in this cohort received prophylactic NIV immediately after extubation. Absence of prophylactic NIV does not make the composite endpoint equivalent to reintubation alone: among the 9 patients with composite extubation failure, 7 (77.8%) were reintubated (3 after a failed NIV trial, 4 directly), whereas 2 received successful rescue NIV and avoided reintubation. Causes of reintubation were upper airway obstruction/secretions (*n* = 2), respiratory failure/atelectasis (*n* = 4), and cardiovascular instability (*n* = 3). A sensitivity analysis using reintubation alone (7 events among 33 extubated patients) is reported in Supplementary Table S8. ΔEELI (T4–T1) remained associated with reintubation (Firth OR 0.38, 95% CI: 0.14–0.71), and the MLE combined model (T4 RSBI + ΔEELI T4–T1) had an apparent AUC of 0.890 (95% CI: 0.704–1.000).

### Exploratory early landmark analysis

An exploratory T2 landmark analysis is reported in Supplementary Table S7. Absolute T2 COV was not associated with subsequent SBT failure (OR: 1.03, 95% CI: 0.93–1.15, *P* = 0.57), in line with Table 2. The T1-to-T2 change in COV (ΔCOV T2–T1) was associated with SBT failure (OR: 1.39, 95% CI: 1.10–1.75, *P* = 0.006). T2 GI and ΔEELI (T2–T1) were not significant.

## Discussion

This prospective observational study investigated the dynamic changes in regional ventilation distribution and end-expiratory lung impedance (EELI) using EIT during spontaneous breathing trials (SBT) to explore their associations with weaning and extubation outcomes. Our findings suggest that patients who failed the SBT exhibited progressive ventilation deterioration, characterized by a sustained increase in the Global Inhomogeneity (GI) index, a significant ventral shift in the center of ventilation (COV), and a pronounced spike in the absolute ventral-to-dorsal (V-D) ventilation difference (|ROI V-D|). Furthermore, a progressive decline in ΔEELI was observed in both SBT and extubation failure groups, suggesting that loss of end-expiratory lung aeration during the SBT is an associated physiological pattern in weaning and extubation failure.

### Dynamic ventilation trajectories and SBT failure

The transition from positive pressure ventilation to low-level pressure support during an SBT serves as a physiological “stress test” for the respiratory system (23, 24). In our cohort, patients who failed the SBT exhibited a progressive increase in the GI index and a significant ventral shift in the COV. This dynamic deterioration suggests that SBT failure is associated with worsening ventilation distribution inhomogeneity and regional ventilation redistribution. Our observation of a significant |ROI V-D| spike at T3 (10–20 min) aligns with the VISION study (25), which established that a ventral-to-dorsal difference >20% is a robust early indicator of liberation failure. Such ventrodorsal imbalance often reflects increased ventrodorsal ventilation imbalance, which is consistent with previously reported patterns of regional ventilation redistribution, both of which increase the work of breathing and impair gas exchange (26, 27). These EIT changes were detectable during the SBT and may serve as associated physiological markers before overt clinical signs such as tachypnea or agitation (28, 29).

### Lung de-recruitment as an associated pattern in extubation failure

A critical finding of our study is that regional ventilation parameters (GI, COV, |ROI V-D|) did not significantly differ between extubation success and failure groups, whereas the cumulative loss of EELI (ΔEELI T4–T1) was strongly associated with extubation failure (AUC 0.833). This suggests that post-extubation respiratory failure may be related to loss of end-expiratory lung aeration that occurs during the SBT but does not immediately disrupt regional distribution patterns. This observation extends the multicenter findings of Longhini et al. (30) and aligns with more recent work by He et al. (31), which emphasized that the stability of end-expiratory lung volume is paramount for maintaining post-extubation aeration. The progressive decline in ΔEELI in the extubation failure group is consistent with loss of end-expiratory lung aeration. Notably, the cumulative change ΔEELI (T4-T1), representing the net EELI change over the entire SBT, showed the strongest association with extubation outcomes, suggesting that monitoring cumulative EELI changes during the SBT may provide a volumetric perspective that traditional indices like RSBI lack.

### Clinical implications and synergy with RSBI

Adding T3 GI to T4 RSBI improved model fit for SBT failure (likelihood-ratio *P* = 0.015; optimism-corrected AUC 0.905; Supplementary Table S3). For extubation failure, the combined MLE model had an apparent AUC of 0.875, but with only 9 events this estimate is hypothesis-generating. RSBI summarizes global mechanical efficiency, whereas EIT describes regional ventilation and end-expiratory impedance (6, 32, 33). Whether these complementary signals can guide weaning decisions requires larger studies with standardized SBT and post-extubation management (15, 34).

## Limitations

Several limitations warrant consideration. (1) This was a single-center observational study with a relatively modest sample size (*n* = 52), which limits the generalizability of our findings. Notably, the extubation analysis included only 9 failure events among 33 extubated patients, resulting in an EPV of 3.0 for the combined model. This substantially limits the reliability of the predictive estimates and means that the extubation analysis should be considered exploratory. All predictive findings, particularly those related to extubation outcomes, require validation in larger independent cohorts before any clinical application can be considered. (2) EIT measurements were taken exclusively in a semi-recumbent position; changes in body position can significantly influence ventilation distribution, and our results may not apply to patients in other postures (35). (3) While we integrated EIT with RSBI, we did not include other emerging markers such as diaphragm ultrasound or esophageal pressure, which could provide further insights into the work of breathing and respiratory muscle function. (4) The SBT protocol used pressure support of 5 cmH_2_O with zero PEEP; whether the findings are directly applicable to other SBT techniques (e.g., T-piece or CPAP) warrants further investigation. (5) RSBI measured at T4 in patients who met SBT failure criteria may reflect an event already in progress rather than predict a subsequent one, as respiratory rate is a component of the SBT failure definition. Similarly, EIT measurements obtained at the time of failure may be concurrent with rather than antecedent to the failure event. This distinction is particularly important for interpreting the predictive performance of T4 measurements. Therefore, our findings regarding T4 parameters should be interpreted as associations with SBT failure rather than definitive early prediction. The T3 parameters (10–20 min), which were obtained before any patient met failure criteria, more directly address the question of early prediction. Furthermore, the calculation method for T4 EIT parameters differed by outcome: for SBT-success patients, T4 was the mean of the 20–30 min interval, while for SBT-failure patients, T4 was the mean of the last 60 s before termination. This outcome-dependent sampling may introduce bias in group comparisons, as the averaging windows were not uniform across all patients. Although this approach was chosen to capture the physiological state immediately preceding failure, it should be acknowledged as a limitation. 6. EIT measurements were performed using a single electrode belt in a single transverse plane at the 4th–5th intercostal space. This approach provides information only about regional ventilation in that specific plane and does not capture craniocaudal gradients or three-dimensional ventilation distribution. The absence of craniocaudal data limits our ability to comment on whether the observed changes occurred predominantly in caudal-dependent regions, cranial regions, or diffusely. Future studies using volumetric EIT with multiple electrode belts would be valuable to address this question. 7. Although our EIT data were acquired at 50 Hz—providing sufficient temporal resolution for potential pendelluft detection—we did not perform exploratory pendelluft analysis in the current study. Pendelluft, defined as intrapulmonary gas redistribution occurring out of phase with the global respiratory cycle, requires dedicated post-processing algorithms for phase-angle determination between regional impedance changes and synchronized respiratory signals, which were not readily available for all patients in our dataset. Moreover, the methodological standardization for pendelluft quantification remains under development, and there is no universally accepted consensus on parameters such as reference signal selection or threshold definition. Future studies with standardized protocols for raw data storage and synchronized signal acquisition could perform this analysis and may provide additional insights into regional timing discordance during the SBT.8. EELI reflects changes in end-expiratory lung impedance measured in arbitrary units (AU) rather than directly measuring functional residual capacity (FRC) or absolute lung volume. Although changes in EELI are commonly interpreted as reflecting lung de-recruitment or loss of end-expiratory lung aeration, this interpretation is based on the established correlation between EIT-derived impedance changes and lung volume changes, rather than direct volumetric measurement. Therefore, all mechanistic interpretations of EELI changes—including references to “lung de-recruitment,” “loss of lung aeration,” or “functional residual capacity reduction”—should be considered speculative and hypothesis-generating. Future studies incorporating concurrent FRC measurements or quantitative lung imaging (e.g., computed tomography) would be valuable to validate the relationship between EELI changes and actual lung volume changes during SBT.

## Conclusions

In this prospective exploratory cohort, patients who failed the spontaneous breathing trial showed different temporal trajectories of regional tidal ventilation distribution, including increasing spatial inhomogeneity and ventrodorsal asymmetry. Among patients who completed the SBT, a greater reduction in end-expiratory lung impedance was associated with adverse post-extubation outcomes. These findings require validation in larger cohorts with standardized post-extubation management and require validation of ventilation-perfusion mismatch or a specific mechanism of regional aeration loss.

## Data Availability

The original contributions presented in the study are included in the article/Supplementary Material, further inquiries can be directed to the corresponding author.
