# Supplementary material for: Dynamic changes in regional ventilation distribution and end-expiratory lung impedance using electrical impedance tomography: associations with weaning and extubation outcomes
Source: Front Med (Lausanne). 2026 Oct 7;13:1904503. doi: 10.3389/fmed.2026.1904503 (PMC13683595; doi:10.3389/fmed.2026.1904503)
Supplement: Supplementary file 1 [file Datasheet1.docx]

**Supplementary Material (Tables S1–S8; Figures S1–S9)**

**Dynamic Changes in Regional Ventilation Distribution and End-Expiratory Lung Impedance Using Electrical Impedance Tomography: Associations with Weaning and Extubation Outcomes**

Minjia Wang, Changyun Zhao, Zhiheng Hao, Liang Wu, Wenchao Mao, Jiaping Zhao

Department of Critical Care Medicine, Zhejiang Hospital, Hangzhou, Zhejiang, China

# **Contents**

1. Supplementary Methods
2. Table S1. Bootstrap internal validation of the MLE prediction models
3. Table S2. Coefficients of the MLE prediction models
4. Table S3. Incremental value of EIT over RSBI
5. Table S4. Valid EIT measurements and distribution of SBT failure times
6. Table S5. Change-score analysis for COV (ΔCOV from baseline)
7. Table S6. Univariable and multivariable logistic/ROC analysis at all time points
8. Table S7. Exploratory early landmark analysis (T2)
9. Table S8. Firth association estimates and reintubation-only sensitivity analysis
10. Figure S1. Calibration of MLE prediction models for SBT failure
11. Figure S2. Calibration of MLE prediction models for extubation failure
12. Figure S3. ROC curves from the MLE prediction models
13. Figure S4. GI index during the SBT according to SBT outcome
14. Figure S5. Center of ventilation during the SBT according to SBT outcome
15. Figure S6. |ROI V-D| during the SBT according to SBT outcome
16. Figure S7. GI index during the SBT according to extubation outcome
17. Figure S8. Center of ventilation during the SBT according to extubation outcome
18. Figure S9. |ROI V-D| during the SBT according to extubation outcome

# **Supplementary Methods**

Two complementary logistic frameworks were used and are kept distinct. Firth penalized logistic regression was used to estimate associations (odds ratios) when the number of events was small, particularly for extubation failure (9 composite events among 33 extubated patients). Profile-likelihood 95% confidence intervals are reported. A separately specified unpenalized maximum-likelihood (MLE) logistic model with the same predictors was used as the prediction model for discrimination (AUC), Brier score, calibration, sensitivity/specificity, nested likelihood-ratio tests, and Harrell bootstrap internal validation (1,000 resamples, seed = 42). Apparent AUCs, optimism-corrected AUCs, bootstrap confidence intervals, and the coefficients in Tables S1–S3 correspond to this MLE prediction model and should not be interpreted as Firth estimates. Firth association estimates for extubation are reported in Table S8.

Calibration used logistic recalibration (intercept with slope fixed at 1, and an intercept-plus-slope model). Sensitivity and specificity were calculated at the Youden threshold of the apparent predicted probabilities, with bootstrap 95% CIs. In-sample calibration intercepts and slopes near 0/1 are expected for MLE models evaluated on the development sample; optimism-corrected AUC and Brier score are therefore emphasized.

T4 calculation. For SBT-success patients (n = 33), all of whom completed the 30-minute trial, T4 was the mean impedance over the entire 20–30 min window (10 minutes), matching the duration used for T2 (0–10 min) and T3 (10–20 min). For SBT-failure patients (n = 19), all of whom failed between 26 and 30 min, T4 was the mean impedance during the last 60 seconds before SBT termination (the cut-off value). The available averaging window therefore ranged from 60 seconds (failure at 26 min) to 180 seconds (failure at 30 min), with a mean of 108 ± 45 seconds. This outcome-dependent difference in averaging windows is a limitation of T4 comparisons. No imputation or last-observation-carried-forward was used. Valid n at each time point and the failure-time distribution are given in Table S4. Extubation failure in the primary analysis was the composite of rescue NIV and/or reintubation within 48 h. The reintubation-only sensitivity analysis recoded the two patients with successful rescue NIV who avoided reintubation (manuscript Table 4, patients 4 and 5) as non-events, leaving 7 reintubation events among 33 extubated patients. No patient received prophylactic NIV.

Outcome coding. Every logistic model in this supplement (Tables S1–S3 and S6–S8) codes 1 = SBT failure or extubation failure and 0 = success. OR > 1 therefore indicates higher odds of failure, which matches the descriptive data (for example, higher T4 RSBI in SBT failure). AUCs for protective predictors (OR < 1) were computed after reversing the predictor so that higher scores indicate higher event risk. Apparent AUCs, optimism-corrected AUCs, Brier scores, calibration metrics, and coefficients in Tables S1–S3 and Figures S1–S3 belong to unpenalized MLE prediction models. Firth penalized odds ratios are reported only in Table S8.

# **Table S1. Bootstrap internal validation of the MLE prediction models**

All quantities are from unpenalized MLE logistic regression (not Firth). Optimism-corrected AUC = apparent AUC − mean Harrell bootstrap optimism. N is the number of observations included in each model. Prespecified predictors: SBT failure — T4 RSBI and T3 GI; extubation failure — T4 RSBI and ΔEELI T4–T1.

| **Outcome** | **Model** | **Stated model** | **Predictors** | **N** | **Events** | **Apparent AUC (95% CI)** | **Optimism** | **Optimism-corrected AUC** | **Brier score** | **Calibration intercept (slope=1)** | **Calibration slope** | **Sensitivity (bootstrap 95% CI)** | **Specificity (bootstrap 95% CI)** |
| --- | --- | --- | --- | --- | --- | --- | --- | --- | --- | --- | --- | --- | --- |
| SBT failure | RSBI only | MLE prediction model | T4 RSBI | 52 | 19 | 0.874 (0.741-0.976) | 0.001 | 0.873 | 0.104 | 0.0 | 1.0 | 0.789 (0.562-0.923) | 0.909 (0.696-1.000) |
| SBT failure | EIT only | MLE prediction model | T3 GI | 52 | 19 | 0.813 (0.665-0.941) | 0.001 | 0.812 | 0.176 | 0.0 | 1.0 | 0.789 (0.000-0.923) | 0.788 (0.429-1.000) |
| SBT failure | Combined (RSBI + EIT) | MLE prediction model | T4 RSBI + T3 GI | 52 | 19 | 0.923 (0.840-0.986) | 0.019 | 0.905 | 0.093 | 0.0 | 1.0 | 0.737 (0.538-0.957) | 0.970 (0.839-1.000) |
| Extubation failure | RSBI only | MLE prediction model | T4 RSBI | 33 | 9 | 0.567 (0.356-0.760) | 0.04 | 0.527 | 0.194 | 0.0 | 1.0 | 1.000 (0.000-1.000) | 0.333 (0.000-1.000) |
| Extubation failure | EIT only | MLE prediction model | ΔEELI T4–T1 | 33 | 9 | 0.833 (0.621-1.000) | 0.003 | 0.83 | 0.118 | -0.0 | 1.0 | 0.778 (0.111-1.000) | 0.958 (0.818-1.000) |
| Extubation failure | Combined (RSBI + EIT) | MLE prediction model | T4 RSBI + ΔEELI T4–T1 | 33 | 9 | 0.875 (0.720-0.991) | 0.02 | 0.855 | 0.112 | -0.0 | 1.0 | 0.778 (0.249-1.000) | 0.917 (0.800-1.000) |

Abbreviations: AUC, area under the receiver operating characteristic curve; CI, confidence interval; COV, center of ventilation; ΔCOV, change in COV from T1; ΔEELI, change in end-expiratory lung impedance; EIT, electrical impedance tomography; GI, global inhomogeneity index; LRT, likelihood-ratio test; MLE, maximum-likelihood estimation; NIV, noninvasive ventilation; OR, odds ratio; |ROI V-D|, absolute ventrodorsal ventilation difference; RSBI, rapid shallow breathing index; SBT, spontaneous breathing trial.

# **Table S2. Coefficients of the MLE prediction models**

Intercepts and coefficients belong to the same unpenalized MLE prediction models used for AUC, Brier score, calibration, and bootstrap internal validation (Table S1). They are not Firth association estimates (see Table S8).

| **Outcome** | **Model** | **Stated model** | **Term** | **Coefficient** | **SE** | **OR (95% CI)** | **P value** |
| --- | --- | --- | --- | --- | --- | --- | --- |
| SBT failure | RSBI only | MLE prediction model | Intercept | -7.528 | 2.079 | — | <0.001 |
| SBT failure | RSBI only | MLE prediction model | T4 RSBI | 0.158 | 0.049 | 1.172 (1.065-1.289) | 0.001 |
| SBT failure | EIT only | MLE prediction model | Intercept | -11.362 | 4.234 | — | 0.007 |
| SBT failure | EIT only | MLE prediction model | T3 GI | 0.232 | 0.09 | 1.261 (1.058-1.503) | 0.010 |
| SBT failure | Combined (RSBI + EIT) | MLE prediction model | Intercept | -20.322 | 7.028 | — | 0.004 |
| SBT failure | Combined (RSBI + EIT) | MLE prediction model | T4 RSBI | 0.195 | 0.066 | 1.215 (1.068-1.383) | 0.003 |
| SBT failure | Combined (RSBI + EIT) | MLE prediction model | T3 GI | 0.244 | 0.112 | 1.276 (1.025-1.589) | 0.029 |
| Extubation failure | RSBI only | MLE prediction model | Intercept | -2.975 | 2.174 | — | 0.171 |
| Extubation failure | RSBI only | MLE prediction model | T4 RSBI | 0.056 | 0.059 | 1.057 (0.942-1.187) | 0.342 |
| Extubation failure | EIT only | MLE prediction model | Intercept | -2.446 | 0.777 | — | 0.002 |
| Extubation failure | EIT only | MLE prediction model | ΔEELI T4–T1 | -1.051 | 0.4 | 0.350 (0.160-0.767) | 0.009 |
| Extubation failure | Combined (RSBI + EIT) | MLE prediction model | Intercept | -6.086 | 3.37 | — | 0.071 |
| Extubation failure | Combined (RSBI + EIT) | MLE prediction model | T4 RSBI | 0.101 | 0.087 | 1.107 (0.933-1.312) | 0.243 |
| Extubation failure | Combined (RSBI + EIT) | MLE prediction model | ΔEELI T4–T1 | -1.07 | 0.406 | 0.343 (0.155-0.761) | 0.009 |

# **Table S3. Incremental value of EIT over RSBI (nested MLE prediction models)**

Prespecified comparison of an RSBI-only MLE model versus an RSBI-plus-EIT MLE model. The likelihood-ratio test assesses improvement in model fit. ΔAUC is Combined minus RSBI, with a bootstrap 95% CI. Lower Brier scores indicate better overall accuracy.

| **Outcome** | **Stated model** | **Base model** | **Full model** | **N (events)** | **LR chi-square (df)** | **LR P value** | **AUC RSBI (optimism-corrected)** | **AUC Combined (optimism-corrected)** | **Delta AUC (95% CI)** | **Delta AUC optimism-corrected** | **Brier RSBI** | **Brier Combined** | **Delta Brier (Combined-RSBI)** |
| --- | --- | --- | --- | --- | --- | --- | --- | --- | --- | --- | --- | --- | --- |
| SBT failure | Nested MLE prediction models | T4 RSBI | T4 RSBI + T3 GI | 52 (19) | 5.919 (1) | 0.015 | 0.874 (0.870) | 0.923 (0.899) | 0.049 (0.004-0.172) | 0.029 | 0.104 | 0.093 | -0.011 |
| Extubation failure | Nested MLE prediction models | T4 RSBI | T4 RSBI + ΔEELI T4–T1 | 33 (9) | 13.941 (1) | <0.001 | 0.567 (0.530) | 0.875 (0.862) | 0.308 (0.061-0.491) | 0.333 | 0.194 | 0.112 | -0.082 |

# **Table S4. Valid EIT measurements at each time point and distribution of SBT failure times**

Part A. Valid EIT measurements by time point (N = 52).

| **Time point** | **Description** | **Patients with valid measurements (n)** | **Percentage (%)** |
| --- | --- | --- | --- |
| T1 | 10 min before SBT initiation | 52 | 100.0 |
| T2 | 0–10 min after SBT initiation | 52 | 100.0 |
| T3 | 10–20 min after SBT initiation | 52 | 100.0 |
| T4 | 20–30 min after SBT initiation | 52 | 100.0 |

Note for Part A: For T4 (20–30 min), patients who failed before 30 minutes had their last valid measurement before termination used as the representative T4 value (cut-off value at the time of SBT failure).

Part B. Distribution of SBT failure times (n = 19).

| **Failure time (minutes)** | **Number of patients (n)** | **Percentage of failure group (%)** | **Cumulative percentage (%)** |
| --- | --- | --- | --- |
| 26 | 3 | 15.8 | 15.8 |
| 27 | 6 | 31.6 | 47.4 |
| 28 | 5 | 26.3 | 73.7 |
| 29 | 3 | 15.8 | 89.5 |
| 30 | 2 | 10.5 | 100.0 |
| Total | 19 | 100.0 | — |

Notes: (a) No imputation, extrapolation, or last-observation-carried-forward was used. (b) The SBT was terminated immediately upon meeting predefined failure criteria. (c) SBT success n = 33; SBT failure n = 19.

Abbreviations: AUC, area under the receiver operating characteristic curve; CI, confidence interval; COV, center of ventilation; ΔCOV, change in COV from T1; ΔEELI, change in end-expiratory lung impedance; EIT, electrical impedance tomography; GI, global inhomogeneity index; LRT, likelihood-ratio test; MLE, maximum-likelihood estimation; NIV, noninvasive ventilation; OR, odds ratio; |ROI V-D|, absolute ventrodorsal ventilation difference; RSBI, rapid shallow breathing index; SBT, spontaneous breathing trial.

# **Table S5. Change-score analysis for COV (ΔCOV from baseline)**

ΔCOV = COV at Tx − COV at T1. Positive values indicate a dorsal shift; negative values indicate a ventral shift. P values are for the within-group change from baseline.

| **Time point** | **Group** | **Mean ΔCOV (%)** | **95% CI** | **P value** |
| --- | --- | --- | --- | --- |
| T2 − T1 | Success (n=33) | +0.88 | −0.52 to 2.28 | 0.218 |
|  | Failure (n=19) | +6.32 | 3.81 to 8.83 | <0.001 |
| T3 − T1 | Success (n=33) | −0.82 | −2.43 to 0.79 | 0.312 |
|  | Failure (n=19) | −0.63 | −1.12 to −0.14 | 0.014 |
| T4 − T1 | Success (n=33) | −1.64 | −3.66 to 0.38 | 0.108 |
|  | Failure (n=19) | −0.68 | −1.21 to −0.15 | 0.013 |

P < 0.05 was considered statistically significant.

# **Table S6. Univariable and multivariable logistic regression and ROC analysis of EIT parameters at all time points**

Odds ratios are for SBT failure or composite extubation failure (1 = failure). AUCs use 1,000 bootstrap resamples (seed = 42). Youden cut-offs are on the original predictor scale. Combined-model AUCs are from MLE predicted probabilities. Multivariable models (Parts C–D) were restricted to T3 regional-ventilation parameters and ΔEELI terms with univariable P < 0.10, matching the specification underlying main-text Table 3; T1/T2/T4 GI, COV, and |ROI V-D| are reported as univariable associations only. Firth-adjusted ORs for the prespecified extubation dual-predictor model are in Table S8, not Part D.

Part A. Univariable analysis of SBT failure (19 events / 52 patients).

| **Variable** | **Univariate OR (95% CI)** | **P** | **AUC (95% CI)** | **Youden cut-off** |
| --- | --- | --- | --- | --- |
| T1 GI | 0.873 (0.754–1.011) | 0.069 | 0.642 (0.491–0.799) | 47.00 |
| T2 GI | 1.083 (0.954–1.228) | 0.219 | 0.624 (0.421–0.803) | 51.00 |
| T3 GI | 1.261 (1.058–1.503) | 0.010 | 0.813 (0.644–0.944) | 48.00 |
| T4 GI | 1.342 (1.104–1.631) | 0.003 | 0.768 (0.624–0.887) | 48.00 |
| T1 COV | 0.830 (0.723–0.953) | 0.008 | 0.716 (0.557–0.846) | 40.00 |
| T2 COV | 1.031 (0.928–1.146) | 0.565 | 0.553 (0.388–0.712) | 44.00 |
| T3 COV | 0.848 (0.747–0.963) | 0.011 | 0.714 (0.558–0.852) | 39.00 |
| T4 COV | 0.862 (0.758–0.980) | 0.023 | 0.707 (0.541–0.846) | 40.00 |
| T1 \|ROI V-D\| | 0.955 (0.865–1.056) | 0.370 | 0.568 (0.401–0.732) | 10.00 |
| T2 \|ROI V-D\| | 1.036 (0.969–1.108) | 0.303 | 0.514 (0.335–0.692) | 27.00 |
| T3 \|ROI V-D\| | 1.090 (1.025–1.159) | 0.006 | 0.691 (0.519–0.851) | 28.00 |
| T4 \|ROI V-D\| | 1.037 (0.978–1.100) | 0.221 | 0.580 (0.396–0.749) | 32.00 |
| ΔEELI T2–T1 | 0.758 (0.515–1.115) | 0.160 | 0.625 (0.456–0.783) | -0.49 |
| ΔEELI T3–T2 | 0.624 (0.413–0.944) | 0.025 | 0.647 (0.467–0.818) | -2.70 |
| ΔEELI T4–T3 | 0.539 (0.349–0.834) | 0.005 | 0.778 (0.634–0.895) | -0.03 |
| ΔEELI T4–T1 | 0.694 (0.550–0.875) | 0.002 | 0.741 (0.563–0.899) | -3.73 |
| T4 RSBI | 1.172 (1.065–1.289) | 0.001 | 0.874 (0.748–0.979) | 43.90 |
| Combined (T4 RSBI + T3 GI) | — | — | 0.923 (0.829–0.986) | — |

Part B. Univariable analysis of composite extubation failure (9 events / 33 patients).

| **Variable** | **Univariate OR (95% CI)** | **P** | **AUC (95% CI)** | **Youden cut-off** |
| --- | --- | --- | --- | --- |
| T1 GI | 0.877 (0.712–1.079) | 0.215 | 0.641 (0.349–0.885) | 40.00 |
| T2 GI | 1.079 (0.870–1.339) | 0.488 | 0.595 (0.333–0.855) | 50.00 |
| T3 GI | 0.966 (0.776–1.203) | 0.760 | 0.546 (0.310–0.775) | 46.00 |
| T4 GI | 0.901 (0.715–1.136) | 0.379 | 0.604 (0.330–0.843) | 45.00 |
| T1 COV | 0.996 (0.861–1.152) | 0.957 | 0.507 (0.285–0.748) | 47.00 |
| T2 COV | 1.013 (0.879–1.167) | 0.863 | 0.516 (0.279–0.736) | 48.00 |
| T3 COV | 0.998 (0.867–1.149) | 0.979 | 0.512 (0.294–0.748) | 44.00 |
| T4 COV | 0.963 (0.823–1.127) | 0.641 | 0.572 (0.320–0.824) | 40.00 |
| T1 \|ROI V-D\| | 1.021 (0.896–1.163) | 0.758 | 0.539 (0.323–0.741) | 14.00 |
| T2 \|ROI V-D\| | 1.042 (0.894–1.214) | 0.598 | 0.521 (0.299–0.759) | 6.00 |
| T3 \|ROI V-D\| | 0.948 (0.848–1.059) | 0.345 | 0.588 (0.347–0.798) | 12.00 |
| T4 \|ROI V-D\| | 1.049 (0.958–1.148) | 0.303 | 0.583 (0.360–0.802) | 8.00 |
| ΔEELI T2–T1 | 0.374 (0.119–1.176) | 0.092 | 0.660 (0.345–0.926) | -0.48 |
| ΔEELI T3–T2 | 0.326 (0.131–0.811) | 0.016 | 0.799 (0.556–0.961) | -0.43 |
| ΔEELI T4–T3 | 0.488 (0.230–1.034) | 0.061 | 0.711 (0.450–0.913) | -0.38 |
| ΔEELI T4–T1 | 0.350 (0.160–0.767) | 0.009 | 0.833 (0.629–1.000) | -2.24 |
| T4 RSBI | 1.057 (0.942–1.187) | 0.342 | 0.567 (0.356–0.765) | 31.48 |
| Combined (T4 RSBI + ΔEELI T4–T1) | — | — | 0.875 (0.713–0.995) | — |

Part C. Multivariable MLE model for SBT failure (T3 parameters and ΔEELI with univariable P < 0.10).

| **Variable** | **Adjusted OR (95% CI)** | **Adjusted P** |
| --- | --- | --- |
| T3 GI | 1.726 (1.152–2.586) | 0.008 |
| T3 COV | 0.918 (0.729–1.157) | 0.469 |
| T3 \|ROI V-D\| | 1.115 (0.964–1.290) | 0.142 |
| ΔEELI T3–T2 | 0.904 (0.406–2.012) | 0.804 |
| ΔEELI T4–T3 | 0.367 (0.147–0.916) | 0.032 |
| ΔEELI T4–T1 | 0.738 (0.511–1.066) | 0.106 |

Part D. Multivariable MLE model for composite extubation failure (ΔEELI terms with univariable P < 0.10). These MLE adjusted ORs are shown for completeness; with 9 events they are unstable. The manuscript reports Firth-adjusted ORs for T4 RSBI + ΔEELI T4–T1 in Table 3 note 3 and Table S8.

| **Variable** | **Adjusted OR (95% CI)** | **Adjusted P** |
| --- | --- | --- |
| ΔEELI T2–T1 | 0.298 (0.072–1.236) | 0.095 |
| ΔEELI T3–T2 | 0.343 (0.047–2.523) | 0.293 |
| ΔEELI T4–T3 | 0.710 (0.180–2.799) | 0.625 |
| ΔEELI T4–T1 | 0.612 (0.162–2.314) | 0.469 |

# **Table S7. Exploratory early landmark analysis (T2 parameters for subsequent SBT failure)**

Early EIT parameters at T2 (0–10 min) and the T1-to-T2 EELI and COV changes were evaluated for association with subsequent SBT failure (1 = failure). All 52 patients had valid T2 measurements, and no patient had met SBT failure criteria by T2. These analyses are exploratory. Absolute T2 COV was not associated with subsequent SBT failure (OR 1.031, 95% CI 0.928–1.146, P = 0.565), which is consistent with the T2 between-group contrast in main-text Table 2 (P_diff = 0.561). The T1-to-T2 change in COV (ΔCOV T2–T1) was associated with SBT failure. A previously drafted landmark OR of 0.90 (0.82–0.99) for “T2 COV” could not be reproduced from the analysis file and should not be cited.

| **Variable** | **Univariate OR (95% CI)** | **P** | **AUC (95% CI)** | **Youden cut-off** |
| --- | --- | --- | --- | --- |
| T2 COV | 1.031 (0.928–1.146) | 0.565 | 0.553 (0.388–0.712) | 44.00 |
| T2 GI | 1.083 (0.954–1.228) | 0.219 | 0.624 (0.421–0.803) | 51.00 |
| T2 \|ROI V-D\| | 1.036 (0.969–1.108) | 0.303 | 0.514 (0.335–0.692) | 27.00 |
| ΔEELI T2–T1 | 0.758 (0.515–1.115) | 0.160 | 0.625 (0.456–0.783) | -0.49 |
| ΔCOV T2–T1 | 1.385 (1.096–1.750) | 0.006 | 0.762 (0.565–0.930) | 3.00 |

# **Table S8. Firth association estimates and reintubation-only sensitivity analysis**

Primary composite extubation failure: 9 events / 33 extubated patients. Reintubation only: 7 events / 33 patients, after recoding manuscript Table 4 patients 4 and 5 (successful rescue NIV without reintubation; 63-year-old woman and 93-year-old woman) as non-events. These two patients correspond to the 4th and 5th records in the extubation-failure block of the analysis file. Composite failure and reintubation are clinically distinct outcomes. Part A reports association estimates. Part B reports apparent AUCs from the separately specified MLE prediction model; Firth ORs should not be paired with MLE AUCs as if they came from one fitted model.

Part A. Association estimates (MLE shown alongside Firth for transparency).

| **Outcome** | **Events/N** | **Model** | **Term** | **MLE OR (95% CI)** | **MLE P** | **Firth OR (profile 95% CI)** | **Firth P (Wald)** |
| --- | --- | --- | --- | --- | --- | --- | --- |
| Composite extubation failure | 9/33 | ΔEELI T4–T1 only | ΔEELI T4–T1 | 0.350 (0.160–0.767) | 0.009 | 0.408 (0.171–0.733) | 0.012 |
| Composite extubation failure | 9/33 | T4 RSBI only | T4 RSBI | 1.057 (0.942–1.187) | 0.342 | 1.047 (0.948–1.183) | 0.413 |
| Composite extubation failure | 9/33 | Combined (T4 RSBI + ΔEELI T4–T1) | T4 RSBI | 1.107 (0.933–1.312) | 0.243 | 1.078 (0.951–1.296) | 0.312 |
| Composite extubation failure | 9/33 | Combined (T4 RSBI + ΔEELI T4–T1) | ΔEELI T4–T1 | 0.343 (0.155–0.761) | 0.009 | 0.424 (0.179–0.736) | 0.012 |
| Reintubation only | 7/33 | ΔEELI T4–T1 only | ΔEELI T4–T1 | 0.308 (0.124–0.765) | 0.011 | 0.380 (0.138–0.707) | 0.013 |
| Reintubation only | 7/33 | T4 RSBI only | T4 RSBI | 1.048 (0.926–1.186) | 0.457 | 1.037 (0.933–1.182) | 0.539 |
| Reintubation only | 7/33 | Combined (T4 RSBI + ΔEELI T4–T1) | T4 RSBI | 1.093 (0.901–1.327) | 0.367 | 1.063 (0.927–1.314) | 0.449 |
| Reintubation only | 7/33 | Combined (T4 RSBI + ΔEELI T4–T1) | ΔEELI T4–T1 | 0.316 (0.131–0.758) | 0.010 | 0.408 (0.156–0.720) | 0.012 |

Part B. Apparent discrimination of the MLE prediction models.

| **Outcome** | **Events/N** | **Model** | **Apparent AUC (MLE prediction, 95% CI)** |
| --- | --- | --- | --- |
| Composite extubation failure | 9/33 | ΔEELI T4–T1 only | 0.833 (0.629–1.000) |
| Composite extubation failure | 9/33 | T4 RSBI only | 0.567 (0.356–0.765) |
| Composite extubation failure | 9/33 | Combined (T4 RSBI + ΔEELI T4–T1) | 0.875 (0.713–0.995) |
| Reintubation only | 7/33 | ΔEELI T4–T1 only | 0.874 (0.657–1.000) |
| Reintubation only | 7/33 | T4 RSBI only | 0.541 (0.295–0.780) |
| Reintubation only | 7/33 | Combined (T4 RSBI + ΔEELI T4–T1) | 0.890 (0.704–1.000) |

Abbreviations: AUC, area under the receiver operating characteristic curve; CI, confidence interval; COV, center of ventilation; ΔCOV, change in COV from T1; ΔEELI, change in end-expiratory lung impedance; EIT, electrical impedance tomography; GI, global inhomogeneity index; LRT, likelihood-ratio test; MLE, maximum-likelihood estimation; NIV, noninvasive ventilation; OR, odds ratio; |ROI V-D|, absolute ventrodorsal ventilation difference; RSBI, rapid shallow breathing index; SBT, spontaneous breathing trial.

# **Figure S1. Calibration of MLE prediction models for SBT failure**

Calibration of the RSBI-only and combined RSBI + T3 GI maximum-likelihood prediction models. The dashed line indicates perfect calibration. These plots correspond to Table S1, not Firth models.

# **Figure S2. Calibration of MLE prediction models for extubation failure**

Calibration of the RSBI-only and combined RSBI + ΔEELI T4–T1 maximum-likelihood prediction models, corresponding to Table S1.

# **Figure S3. ROC curves from the MLE prediction models**

(A) SBT failure: T4 RSBI, T3 GI, and combined T4 RSBI + T3 GI. (B) Extubation failure: T4 RSBI, ΔEELI T4–T1, and combined T4 RSBI + ΔEELI T4–T1. AUCs with bootstrap 95% CIs are from the unpenalized MLE prediction models. Main-text Figure 6 shows the same comparison in a more compact form; Figure S3 is retained to display confidence intervals in the legend.

# **Figure S4. GI index during the SBT according to SBT outcome**

Box plots of the GI index at T1–T4 in patients with SBT success versus SBT failure. Between-group differences after Holm–Bonferroni correction are reported in main-text Table 2.

# **Figure S5. Center of ventilation during the SBT according to SBT outcome**

Box plots of COV at T1–T4 by SBT outcome. Between-group differences after Holm–Bonferroni correction are reported in main-text Table 2. Change-score analysis is given in Table S5.

# **Figure S6. |ROI V-D| during the SBT according to SBT outcome**

Box plots of the absolute ventrodorsal ventilation difference at T1–T4 by SBT outcome. Between-group differences after Holm–Bonferroni correction are reported in main-text Table 2.

# **Figure S7. GI index during the SBT according to extubation outcome**

Box plots of the GI index at T1–T4 in patients with extubation success versus composite extubation failure. Between-group differences were not statistically significant at any time point (all P > 0.05).

# **Figure S8. Center of ventilation during the SBT according to extubation outcome**

Box plots of COV at T1–T4 by extubation outcome. Between-group differences were not statistically significant at any time point (all P > 0.05).

# **Figure S9. |ROI V-D| during the SBT according to extubation outcome**

Box plots of the absolute ventrodorsal ventilation difference at T1–T4 by extubation outcome. Between-group differences were not statistically significant at any time point (all P > 0.05).
